# LINC00629 protects osteosarcoma cell from ER stress-induced apoptosis and facilitates tumour progression by elevating KLF4 stability

**DOI:** 10.1186/s13046-022-02569-x

**Published:** 2022-12-20

**Authors:** Yuan Wang, Shuo Zheng, Jian Han, Na Li, Renchen Ji, Xiaodong Li, Chuanchun Han, Wenzhi Zhao, Lu Zhang

**Affiliations:** 1 The Second Affiliated Hospital & Institute of Cancer Stem Cell, Dalian Medical University, Dalian, Liaoning 116044 People’s Republic of China; 2 Department of Orthopedics, The Third People’s Hospital of Dalian, Dalian Medical University, Dalian, Liaoning 116033 People’s Republic of China; 3 National-Local Joint Engineering Research Center for Drug-Research and Development (R&D) of Neurodegenerative Diseases, Dalian Medical University, Dalian, 116044 People’s Republic of China

**Keywords:** LINC00629, Osteosarcoma, KLF4, LAMA4

## Abstract

**Background:**

Escaping from ER stress-induced apoptosis plays an important role in the progression of many tumours. However, its molecular mechanism in osteosarcoma remains incompletely understood.

**Methods:**

The molecular mechanism was investigated using RNA sequencing, qRT–PCR and Western blot assays. The relationship between LINC00629 and KLF4 was investigated using RNA pulldown and ubiquitylation assays. The transcriptional regulation of laminin subunit alpha 4 (LAMA4) by KLF4 was identified using bioinformatic analysis, a luciferase assay, and a chromatin immunoprecipitation assay.

**Results:**

Here, we demonstrated that LINC00629 was increased under ER stress treatment. Elevated LINC00629 inhibited ER stress-induced osteosarcoma cell apoptosis and promoted clonogenicity and migration in vitro and in vivo. Further mechanistic studies indicated that LINC00629 interacted with KLF4 and suppressed its degradation, which led to a KLF4 increase in osteosarcoma. In addition, we also found that KLF4 upregulated LAMA4 expression by directly binding to its promoter and that LINC00629 inhibited ER stress-induced apoptosis and facilitated osteosarcoma cell clonogenicity and metastasis by activating the KLF4-LAMA4 pathway.

**Conclusion:**

Collectively, our data indicate that LINC00629 is a critical long noncoding RNA (lncRNA) induced by ER stress and plays an oncogenic role in osteosarcoma cell by activating the KLF4-LAMA4 axis.

**Supplementary Information:**

The online version contains supplementary material available at 10.1186/s13046-022-02569-x.

## Introduction

Osteosarcoma is one of the most common bone malignancies that often occurs in adolescents. In recent years, the 5-year survival rates of patients with localized osteosarcoma have remained at 60–70% due to surgery together with multiagent chemotherapy [1]. However, after metastases development, the 5-year survival rate is still less than 20% [2–4]. Thus, metastasis remains a major obstacle to the treatment of osteosarcoma.

Tumour metastasis is a multistep process that releases cancer cells from the primary organ to colonize distant organs, where they can form another tumour [5]. When tumours metastasise, the cells undergo diverse microenvironments, including nutrient deprivation and hypoxia, resulting in endoplasmic reticulum (ER) stress [6, 7]. Previous studies indicated that tumour cells can develop an adaptive mechanism to ER stress and escape from ER stress-induced apoptosis, which later contributes to tumorigenesis and metastasis [8–10]. Therefore, uncovering the molecular mechanisms by which cells escape ER stress-induced apoptosis is important for developing new strategies for preventing osteosarcoma metastasis.

Long noncoding RNAs (lncRNAs) are a class of nonprotein coding transcripts longer than 200 nucleotides and are poorly conserved [11]. Based on their genomic location, LncRNAs can be grouped into different classes of transcripts, including intergenic lncRNAs (lincRNAs), intronic lncRNAs and antisense lncRNAs [12]. Accumulating evidence indicates that lncRNAs play an important role in osteosarcoma [13]. For example, lncRNA KCNQ1OT1 promoted osteosarcoma growth by enhancing aerobic glycolysis [14]. lncRNA CCAT2 promoted osteosarcoma cell proliferation and invasion [15]. LINC00161 increased chemosensitivity by regulating the miR-645-IFIT2 axis in osteosarcoma cells [16]. Our previous studies suggested that ZBTB7A suppressed LINC00473 expression and inhibited cisplatin-induced apoptosis [17] and ZBTB7A could impair ER stress-induced apoptosis by downregulating GAS5 expression [18]. However, the lncRNAs that facilitate osteosarcoma escape from ER stress-induced apoptosis are still not well understood.

In this study, we found that LINC00629 was upregulated in response to ER stress treatment. Elevated LINC00629 suppressed osteosarcoma cell apoptosis and facilitated tumorigenesis and metastasis in vitro and in vivo. Subsequently, we found that LINC00629 interacted with KLF4 and suppressed its degradation, which led to an increase in KLF4 in 143B and MNNG/HOS cells. In addition, we also found that KLF4 upregulated LAMA4 expression by directly binding to its promoter. LINC00629 inhibited ER stress-induced apoptosis and facilitated osteosarcoma cell tumorigenesis and metastasis by activating the KLF4-LAMA4 pathway. Collectively, our data indicate that LINC00629 is a critical lncRNA induced by ER stress and plays an oncogenic role in osteosarcoma cell.

## Methods

### Cell culture and reagents

Human osteosarcoma cell 143B, MG63 and U2OS were maintained in Dulbecco’s modified Eagle medium (DMEM). MNNG/HOS cells were cultured in Minimum Essential Medium (MEM). These media were supplemented with 10% foetal bovine serum (FBS), 2 mM L-glutamine, penicillin (100 U/ml), and streptomycin (100 μg/ml) in a humidified atmosphere of 5% CO2 maintained at 37 °C. SJSA-1 cells were cultured in Roswell Park Memorial Institute (RPMI) 1640 with 10% foetal bovine serum (FBS), 2 mM L-glutamine, penicillin (100 U/ml), and streptomycin (100 μg/ml) in a humidified atmosphere of 5% CO2 maintained at 37 °C. All the cells were cultured in the cell culture dishes/plates which were obtained from NEST Biotechnology Co. Ltd. (Wuxi, China). The following antibodies were used in this study: antibodies against GAPDH (Santa Cruz Biotechnology, Dallas, TX, USA; SC-25778, 1:1000), PARP (Santa Cruz Biotechnology, SC-8007, 1:1000), GRP78 (Santa Cruz Biotechnology, SC-13968, 1:1000 for WB), KLF4 (Cell Signaling Technology, #12173S, 1:500), and LAMA4 (R&D Systems, AF7340, 1:200), LINC00629 SMARTPool (horizon), Tunicamycin (TM, Lot:T7765) and thapsigargin (TG, Lot:T9033) were purchased from Sigma Chemical Co. They were dissolved in DMSO and developed in stock solutions of 3 mmol/L and 1 mmol/L for TG. Cells were treated with 3 μmol/L TM or 1 μmol/L at the indicated times.

### Lentivirus packaging and infection

To generate the lentiviral shRNA constructs against human LINC00629, KLF4 and LAMA4, the target sequences were cloned into pLKO.1-puro vector. The shRNA sequences were listed in Supplementary Table 1. To generate the lentiviral expression vector for LINC00629, it was constructed into a pCDH vector. To establish a stable cell line, the pLKO.1 vector, pVSVG, pREV and pGAG or pCDH vector, psPax2, and pMD2G were contransfected into HEK293T cells. Six hours after transfection, we changed the media to 8 ml DMEM/20% FBS per 10 cm plate and incubated additional 48 hours to generate lentivirus. 48 hrs after post-media change, we harvested the viral supernatant and spun down at1500rpm for 10 min and collected the supernatant. Then, we added the supernatant into the osteosarcoma cells as indicated. Twenty-four hours after infection, osteosarcoma cells were cultured in medium containing 2.5 mg/ml puromycin for the selection of stable clones. The knockdown or overexpression efficiency was evaluated by Western blot and qRT-PCR.

### Quantitative reverse transcription PCR (qRT-PCR)

Total RNA was isolated using Trizol (Invitrogen). One microgram of total RNA was used to synthesize cDNA using the PrimeScriptTM RT reagent kit (Takara, RR047A) according to the manufacturer’s instructions. The primers were listed in Supplementary Table 2. The expression levels of these genes were normalized to those of β-actin. Changes in gene expression were determined using the 2^−ΔΔCT^ method.

### Cell viability and colony formation assays

The cell viabilities of osteosarcoma cells were determined by CCK8 assay. In brief, 143B and MNNG/HOS cells as indicated were seeded at a density of 3000 cells/well in 96-well plates and incubated overnight. The next day, the cells were treated with TM or TG at the indicated concentrations for 36 or 48 h. The absorbance was measured with a spectrometer.

For the colony formation assay, the indicated 143B and MNNG/HOS cells were seeded into a 6-well plate (3000 cell per well) and cultured at 37 °C in a 5% CO_2_ incubator for 1 weeks. Growth media were replenished every 48 h during a 1-week period. Then, Cell colonies were fixed with methanol for 15 min. Following PBS washes, cells were stained with 0.1% crystal violet for 15 min. Images of cell colonies were captured using the Bio-Rad ChemiDoc XRS+ system and quantified with the ImageJ programme.

### Chromatin immunoprecipitation (ChIP) assay

MNNG/HOS Cells were crosslinked with 1% formaldehyde for 10 min at room temperature. The ChIP assay was performed according to the manufacturer’s instructions using the anti-KLF4 antibody and a kit (EZ-ChIP, 17–371 Millipore, Merck KGaA, Darmstadt Germany). Anti-rabbit IgG was used as the control. The bound DNA fragments were eluted and amplified by PCR. PCR products were separated by gel electrophoresis.

### RNA sequencing analysis and label-free quantitative proteomics

MNNG/HOS cells were treated with 3 μM TM for 36 h. Then the cells were collected and transported to BioMaker. RNA extraction, library construction, sequencing and data analysis were performed by BioMaker, Beijing, China.

For label-free quantitative proteomics, 10^6^ MNNG/HOS cells with or without LINC00629 knockdown were collected and transported to Jingjie PTM Biolab, Hongzhou, China.

### Protein half-life assay and in vivo KLF4 ubiquitylation assay

For the KLF4 half-life assay, MNNG/HOS and 143B cells with or without LINC00629 depletion or overexpression were treated with cycloheximide (CHX, Sigma, 10 mg/ml) for the indicated durations before collection and Western blot analysis.

For the KLF4 ubiquitylation assay, HA-ubiquitin was transfected into osteosarcoma cells with or without LINC00629 depletion and overexpression. The cells were then treated with 20 μM MG132 (Calbiochem) for 8 h. These cells were lysed with NP40 lysis buffer and incubated with the indicated primary antibodies. After washing with PBS three times, proteins were released from the beads by boiling in SDS–PAGE sample buffer and analyzed using the anti-Ub antibody.

### RNA pulldown assay

RNA pulldown assays were performed as previously described [19].

### Promoter reporters and dual-luciferase assay

The promoter of LAMA4 and the matching mutant were constructed into a pGL3-basic vector. Luciferase activity was measured in a 1.5-ml Eppendorf tube with a Promega Dual-Luciferases Reporter Assay kit (Promega E1980) according to the manufacturer’s protocol after transfection. Relative Renilla luciferase activity was normalized to firefly luciferase activity. The assay was performed as previously described [19].

### Cell migration assay

Osteosarcoma cell migration assay was conducted and 10,000 cells were seeded in a 24-well Transwell plate with 8-mm polyethylene terephthalate membrane filters (Corning, 3422). The cells resuspended in 200 μL serum-free media were seeded into the upper chamber, and a total of 650 μL complete medium supplemented with 10% FBS was added into the lower chamber. After incubation at 37 °C with 5% CO_2_, the cells that passed through the membrane were fixed with 4% formaldehyde for 30 min and stained with 0.1% crystal violet for 20 min. After wiping off the upper layer of non-migrated or non-invasive cells with a cotton swab, cells were counted by light microscopy.

### Sphere formation assay

Spheres were enriched from osteosarcoma cells with or without LINC00629 knockdown by culturing 1000 cells/mL in serum-free DMEM-F12 medium (Gibco) supplemented with B27 (1:50, Invitrogen) and 20 ng/mL EGF and bFGF. Nontreated tissue culture flasks were used to reduce cell adherence and support growth as undifferentiated tumor spheres. Cells were cultured for 2 weeks, and the number of spheres with a diameter more than 100 μm in each well was counted.

### In vivo tumorigenesis and metastasis assays

Animal research was carried out according to the National Institute of Health Guide for the Care and Use of Laboratory Animals under the approval of the Animal Research Committee of Dalian Medical University. Male nude mice (4–6 weeks old, 18–20 g) were obtained from the SPF Laboratory Animal Center of Dalian Medical University (Dalian, China) and were randomly divided into the indicated groups. The indicated osteosarcoma cells (1 × 10^6^ per mouse) resuspended in 100 μl Phosphate Buffer Saline (PBS) were subcutaneously injected into nude mice. After 10 days, the size of the tumour was measured by Vernier callipers every 2 days and converted to TV according to the following formula: TV (mm^3^) = (a × b^2)/2, where a and b are the maximum and minimum diameters, respectively. All animals were euthanized 18 days after the injection, and the transplanted tumours were removed, weighed and divided into two for further study.

For the in vivo metastasis assay: The indicated osteosarcoma cells (1 × 10^6^ per mouse) resuspended in 100 μl PBS were injected into nude mice through the lateral tail vein. All animals were euthanized 30 days after the injection. The lungs were fixed with 4% formalin and embedded in paraffin blocks. The metastatic lesions were confirmed by histological analysis.

### Bioinformatics analysis

In this study, we used the TNMplot (https://tnmplot.com) database to analyze the RNA expression of LAMA4 in normal(*n* = 564) and osteosarcoma(*n* = 88) tissues [20]. We used the Kaplan–Meier plotter (KM plotter, http://kmplot.com) database to analyze the relationship between LINC00629 or LAMA4 expression and prognoses in the patient with sarcoma.

### Statistics and data analyses

The data were expressed as the mean ± SD, and were statistically evaluated using GraphPad Prism 5. Multiple comparisons between treatment groups and control groups were performed using Dunnett’s least significant difference (LSD) test. Values of *p* < 0.05 were considered statistically significant.

## Results

### LINC00629 is increased by ER stress and suppresses cell apoptosis in osteosarcoma cell

**Fig. 1:**
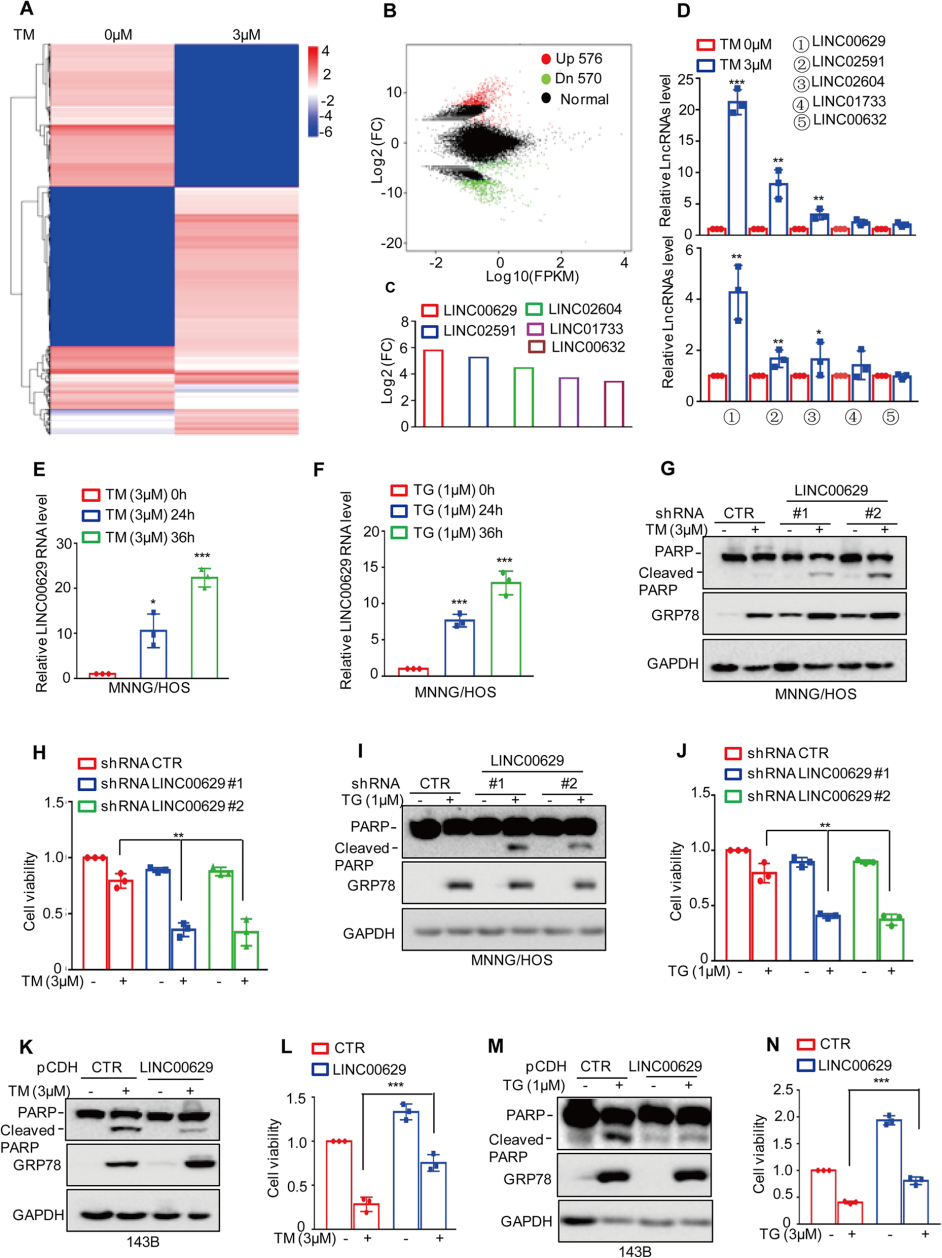
LINC00629 was increased by ER stress and suppressed cell apoptosis (**A**-**B**) MNNG/HOS cells were treated with 3 μM TM for 36 h. The altered LncRNAs were investigated by RNA sequencing analysis. **C** The significantly altered LncRNAs are listed. **D** MNNG/HOS and 143B cells were treated with 3 μM TM for 36 h. The expression levels of the LncRNAs were measured by qRT–PCR in MNNG/HOS (up) and 143B cells (down). **E** MNNG/HOS cells were treated with 3 μM TM for the indicated times. The expression levels of LINC00629 were measured by qRT–PCR. **F** MNNG/HOS cells were treated with 1 μM TG for the indicated times. The expression levels of LINC00629 were measured by qRT–PCR. **G**-**H** MNNG/HOS cells with or without LINC00629 knockdown were treated with or without 3 μM TM for 36 h. Cell apoptosis was detected by Western blot (**G**), and cell viability was analysed by CCK8 assay (**H**). GRP78 was used as the ER stress marker, and GAPDH was used as the loading control. **I**-**J** MNNG/HOS cells with or without LINC00629 knockdown were treated with or without 1 μM TG for 36 h. Cell apoptosis was detected by Western blot (**I**), and cell viability was analysed by CCK8 assay (**J**). (K-L) LINC00629 was overexpressed in 143B cells using lentivirus expressing the pCDH vector, and then the cells were treated with or without 3 μM TM for 48 h. Cell apoptosis was detected by Western blot (**K**), and cell viability was analysed by CCK8 assay (**L**). **M**-**N** 143B cells with or without LINC00629 overexpression were treated with 1 μM TG for 48 h. Cell apoptosis and cell viability were detected by Western blot (**M**) and CCK8 assays (**N**). Data in D, E, F, H, J, L, N were analysed by Student’s t test, *p < 0.05, **p < 0.01, ***p < 0.001

To investigate the lncRNAs altered upon ER stress, the human osteosarcoma cell line MNNG/HOS was treated with 3 μM tunicamycin (TM) to induce pharmacological ER stress, and the cells were then subjected to lncRNA sequencing analysis. As shown in Fig. 1A-B, 567 upregulated and 570 downregulated lncRNAs were obtained (Supplementary Table 3). Among these altered lncRNAs, five significantly changed lncRNAs, LINC00629, LINC02591, LINC02604, LINC01733 and LINC00632, were selected (Fig. 1C). These lncRNAs were then confirmed by qRT–PCR. The result showed that LINC00629 was the most increased among the other significantly upregulated lncRNAs upon TM treatment in MNNG/HOS and 143B cells (Fig. 1D) and was gradually upregulated with increasing TM treatment time (Fig. 1E and Supplementary Fig. 1A).

To further verify that LINC00629 was a ER stress-induced lncRNA, we then treated MNNG/HOS cells with 1 μM thapsigargin (TG), a Ca^2+^-ATPase inhibitor, to induce ER stress, and the expression levels of LINC00629 were analysed by qRT–PCR. We found that LINC00629 was also remarkably increased in response to TG treatment (Fig. 1F). In addition, our subsequent data indicated that sarcoma patients with high LINC00629 expression had short survival times (Supplementary Fig. 1B).

To further investigate the role of LINC00629 in ER stress-induced apoptosis, we first stably knocked down LINC00629 in MNNG/HOS and 143B cells. Compared with those of control group, the expression of LINC00629 in shRNA LINC00629 groups were observably reduced (Supplementary Fig. 1C). These cells were then treated with 3 μM TM or 1 μM TG for 36 h. Western blot and CCK8 assays were performed to investigate the effects of LINC00629 on cell apoptosis and cell viability. Our data indicated that depletion of LINC00629 enhanced ER stress-induced cell apoptosis and promoted a decrease in cell viability (Fig. 1G-J and Supplementary Fig. 1D-E). Whereas, overexpression of LINC00629 decreased cell apoptosis and impaired ER stress-induced cell viability downregulation (Fig. 1K-N and Supplementary Fig. 1F). After that, to exclude off-target effects, the siRNA pool containing four different targeting sequences was used to knock down LINC00629 in MNNG/HOS cells, leading to significant inhibition of LINC00629 (Supplementary Fig. 1G). Consistently, inhibition of LINC00629 promoted ER stress-induced cell apoptosis and decreased cell viability (Supplementary Fig. 1H-I). Subsequently, we further detected the expression of LINC00629 in human osteosarcoma cell lines SJSA-1, U2OS, 143B, MNNG/HOS and MG63. We found that the expression levels of LINC00629 in SJSA-1 cells were lower than the other cells and SJSA-1 cells were sensitive to TM-induced apoptosis (Supplementary Fig. 1 J-K). Elevated LINC00629 inhibited TM-induced SJSA-1cell apoptosis (Supplementary Fig. 1 L).

Taken together, our data suggest that LINC00629 is an ER stress-induced lncRNA and contributes to the adaptation of osteosarcoma cell to ER stress.

### LINC00629 promotes osteosarcoma cell tumorigenesis and metastasis in vitro and in vivo

**Fig. 2:**
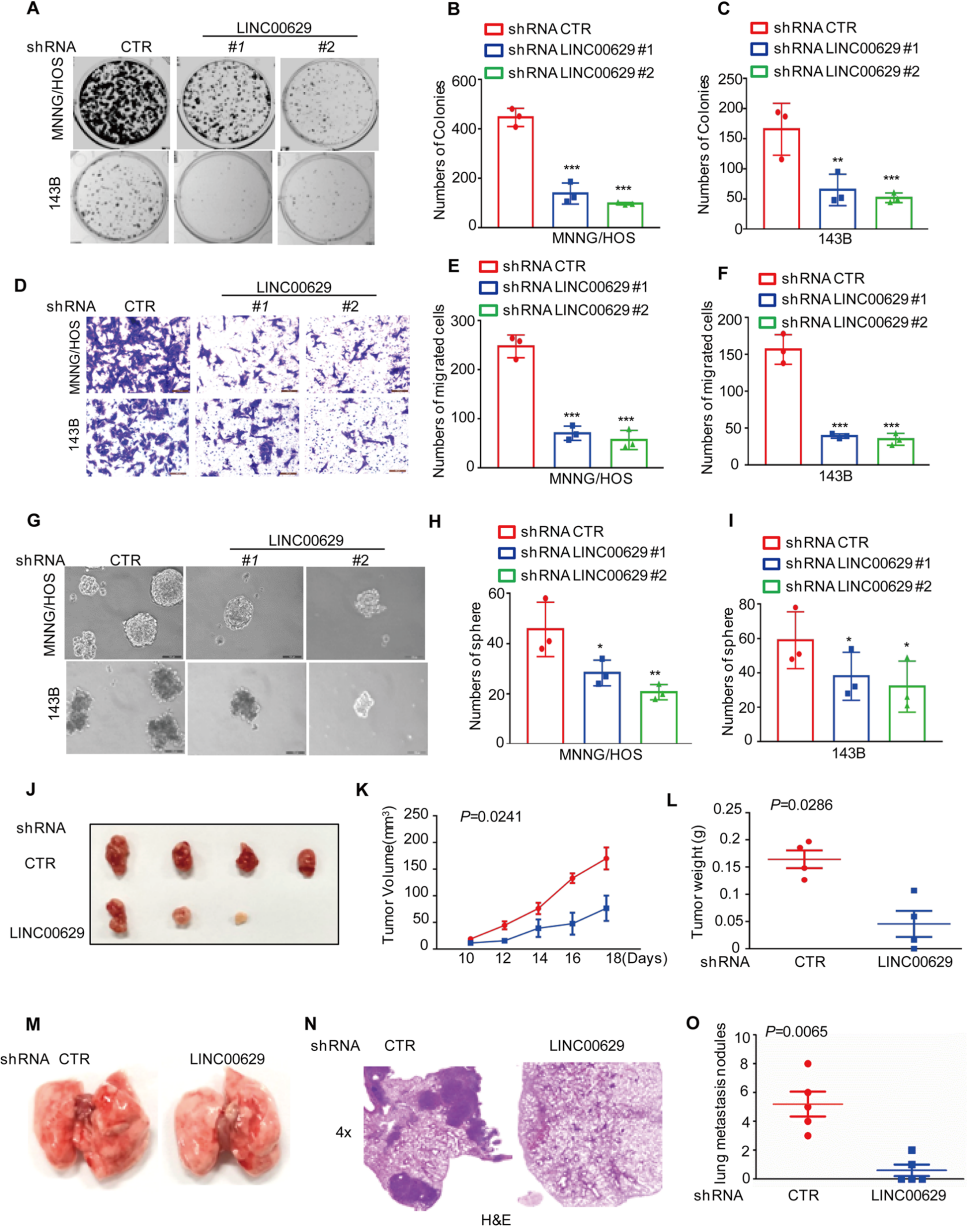
LINC00629 promotes osteosarcoma tumorigenesis and metastasis in vitro and in vivo (**A**-**C**) Osteosarcoma cells (3000 cells/well) with or without LINC00629 depletion were tested for cell growth in the colony formation assay. After 1 week, viable colonies were counted and are shown (**A**). Data are depicted as bar graphs (**B**-**C**). **D**-**F** The migration of the indicated cells was detected by Transwell assays. Representative images of crystal violet-stained culture plates are shown (**D**). Data are depicted as bar graphs (**E**, **F**). **G**-**I** The sphere formation abilities were detected in MNNG/HOS and 143B cells with or without LINC00629 knockdown. Representative images of the spheres are shown (**G**). Data are depicted as bar graphs (**H**-**I**). **J**-**L** MNNG/HOS cells (10^6^ cells per mouse) with or without LINC00629 knockdown were injected subcutaneously into nude mice (*n* = 4). Representative images of xenograft tumours (**J**). The volume (**K**) and weight (**L**) of the tumours were calculated and analysed. **M**-**O** MNNG/HOS cells (10^6^ cells per mouse) with or without LINC00629 knockdown were injected intravenously into nude mice (*n* = 5 per group). Representative images of lung (**M**) and HE (**N**) staining are displayed. Each group of metastatic nodules was assessed (**O**). Data in B, C, E, F, H, I, K, L and O were analysed by Student’s t test, *p < 0.05, **p < 0.01, ***p < 0.001

To further explore the role of LINC00629 in osteosarcoma, colony formation and Transwell assays were performed to measure the effects of LINC00629 on cell clonogenicity and migration. As shown in Fig. 2A-F, depletion of LINC00629 markedly inhibited cell clonogenic potential and migration in MNNG/HOS and 143B cells. In contrast, forced expression of LINC00629 promoted cell clonogenicity and migration (Supplementary Fig. 2A-D). The subsequent sphere formation results suggested that inhibition of LINC00629 impaired the sphere formation capacity of MNNG/HOS and 143B cells (Fig. 2G-I).

To better understand the function of LINC00629 in osteosarcoma cell, we then determined the effects of LINC00629 on tumour growth and metastasis in vivo. To this end, MNNG/HOS cells with or without LINC00629 knockdown were implanted into nude mice. Compared with the control group, knockdown of LINC00629 significantly suppressed tumour formation, as indicated by reduced tumour weights and tumour sizes (Fig. 2J-L). Similarly, LINC00629-depleted cells exhibited significantly decreased lung metastasis abilities at approximately 3 weeks, as indicated by a notable decrease in the number of metastatic lesions and the average surface areas produced by LINC00629-depleted cells (Fig. 2M-O). Collectively, these data indicate that LINC00629 is an important oncogene in MNNG/HOS and 143B cells.

### LINC00629 interacts with KLF4 and enhances its expression in MNNG/HOS and 143B cells

**Fig. 3:**
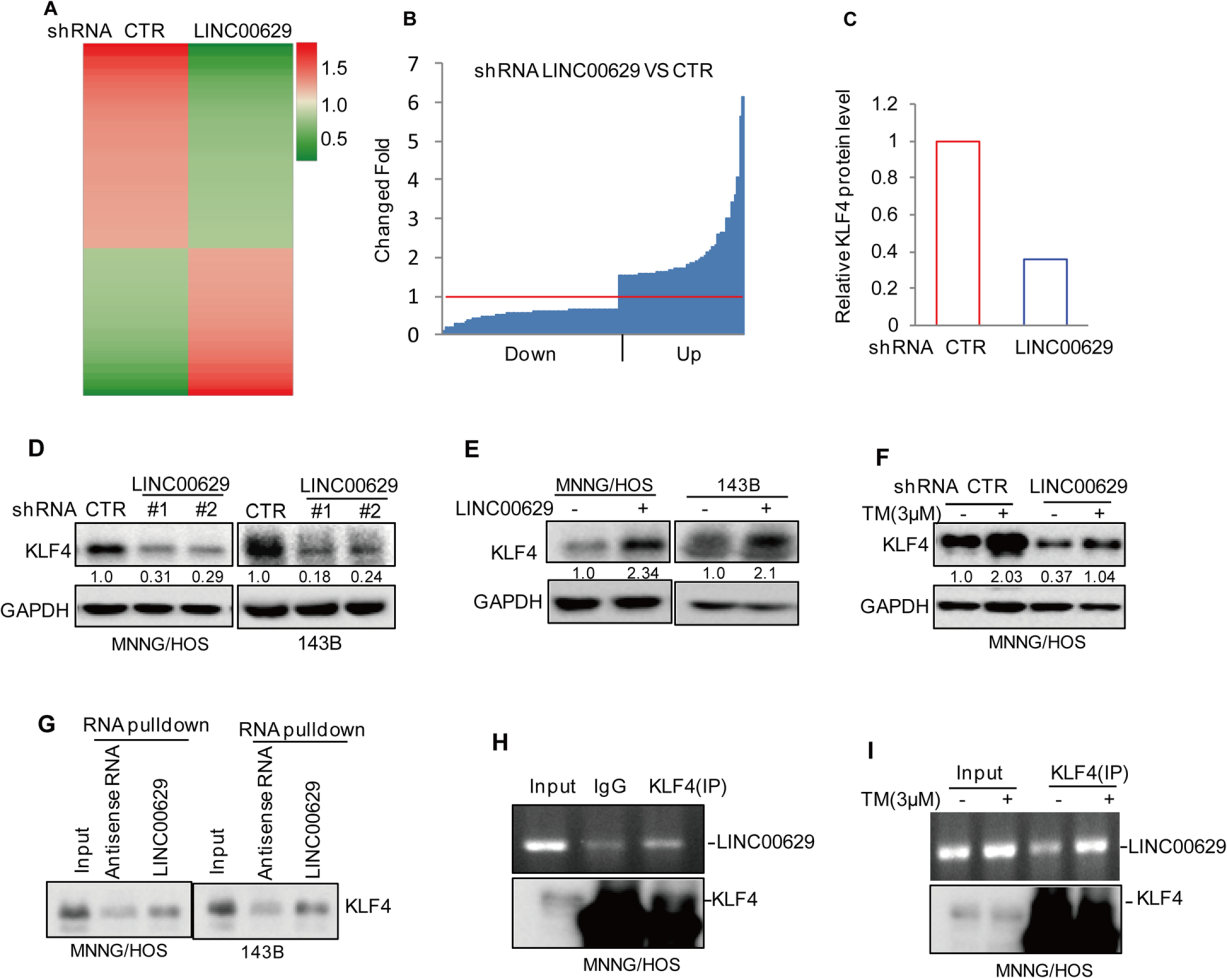
LINC00629 interacts with KLF4 and enhances KLF4 expression in osteosarcoma. **A** The differentially expressed proteins in MNNG/HOS cells with or without LINC00629 knockdown were identified by label-free quantitative proteomics. **B** The sixty-four downregulated and forty-six upregulated proteins are listed. **C** The fold change in KLF4 is listed. **D** The expression levels of KLF4 were detected by Western blot in MNNG/HOS and 143B cells with or without LINC00629 knockdown. Numbers represent the relative intensities of western blot bands of KLF4 to GAPDH. **E** The expression levels of KLF4 were detected by Western blot in MNNG/HOS and 143B cells with or without LINC00629 overexpression. Numbers represent the relative intensities of western blot bands of KLF4 to GAPDH. **F** MNNG/HOS cells with or without LINC00629 knockdown were treated with 3 μM TM for 36 h. The expression levels of KLF4 were detected by Western blot. Numbers represent the relative intensities of western blot bands of KLF4 to GAPDH. **G** Biotin-labelled LINC00629 or antisense RNA was pulled down with KLF4 in whole-cell lysates of MNNG/HOS and 143B cells. KLF4 protein was detected by Western blot. **H** KLF4 antibody (2 μg) was used to coprecipitate with LINC00629 in whole-cell lysates of MNNG/HOS cells. The levels of LINC00629 were detected by RT–PCR, and KLF4 protein levels were analysed by Western blot using a KLF4 antibody. **I** KLF4 antibody was used to coprecipitate with LINC00629 in whole-cell lysates of MNNG/HOS cells with or without 3 μM TM treatment. The levels of LINC00629 were detected by RT–PCR, and KLF4 protein levels were analysed by Western blot using a KLF4 antibody

To elucidate the molecular mechanism whereby LINC00629 inhibited ER stress-induced apoptosis and promoted tumorigenesis in osteosarcoma, we used label-free quantitative proteomics to identify the differentially expressed proteins in MNNG/HOS cells with or without LINC00629 knockdown. Sixty-four downregulated and 56 upregulated genes were observed (Fig. 3A-B). Among these altered genes, we found that KLF4 was significantly downregulated in LINC00629-depleted cells (Fig. 3C). To further verify it, the expression levels of KLF4 were detected in MNNG/HOS and 143B cells with or without LINC00629 knockdown. Consistently, LINC00629 knockdown markedly decreased KLF4 protein levels but did not affect KLF4 mRNA levels (Fig. 3D and Supplementary Fig. 3A-B). Otherwise, elevated LINC00629 increased KLF4 expression (Fig. 3E).

Based the data that LINC00629 was increased in response to ER stress, we thus wanted to know whether LINC00629 contributed to KLF4 increase under ER stress. To this end, we detected the KLF4 expression in MNNG/HOS and 143B cells with or without LINC00629 knockdown under ER stress treatment and observed that KLF4 was significantly increased in response to TM treatment. However, the upregulation of KLF4 was impaired by LINC00629 knockdown (Fig. 3F and Supplementary Fig. 3C).

Considering that LINC00629 facilitated KLF4 protein expression, we wanted to determine whether LINC00629 interacts with KLF4. To test this hypothesis, we carried out an RNA pulldown assay in which biotin-labelled LINC00629 and antisense RNA were synthesized in vitro and incubated with whole-cell lysates of MNNG/HOS and 143B cells. As expected, KLF4 was precipitated by LINC00629 (Fig. 3G). In addition, an RNA immunoprecipitation (RIP) assay was performed and the results indicated that LINC00629 was enriched by the anti-KLF4 antibody relative to IgG, and the enrichment of LINC00629 in the KLF4 immunoprecipitate was increased under ER stress treatment (Fig. 3H-I and Supplementary Fig. 3D-E).

### LINC00629 maintains KLF4 stability and inhibits its degradation

**Fig. 4:**
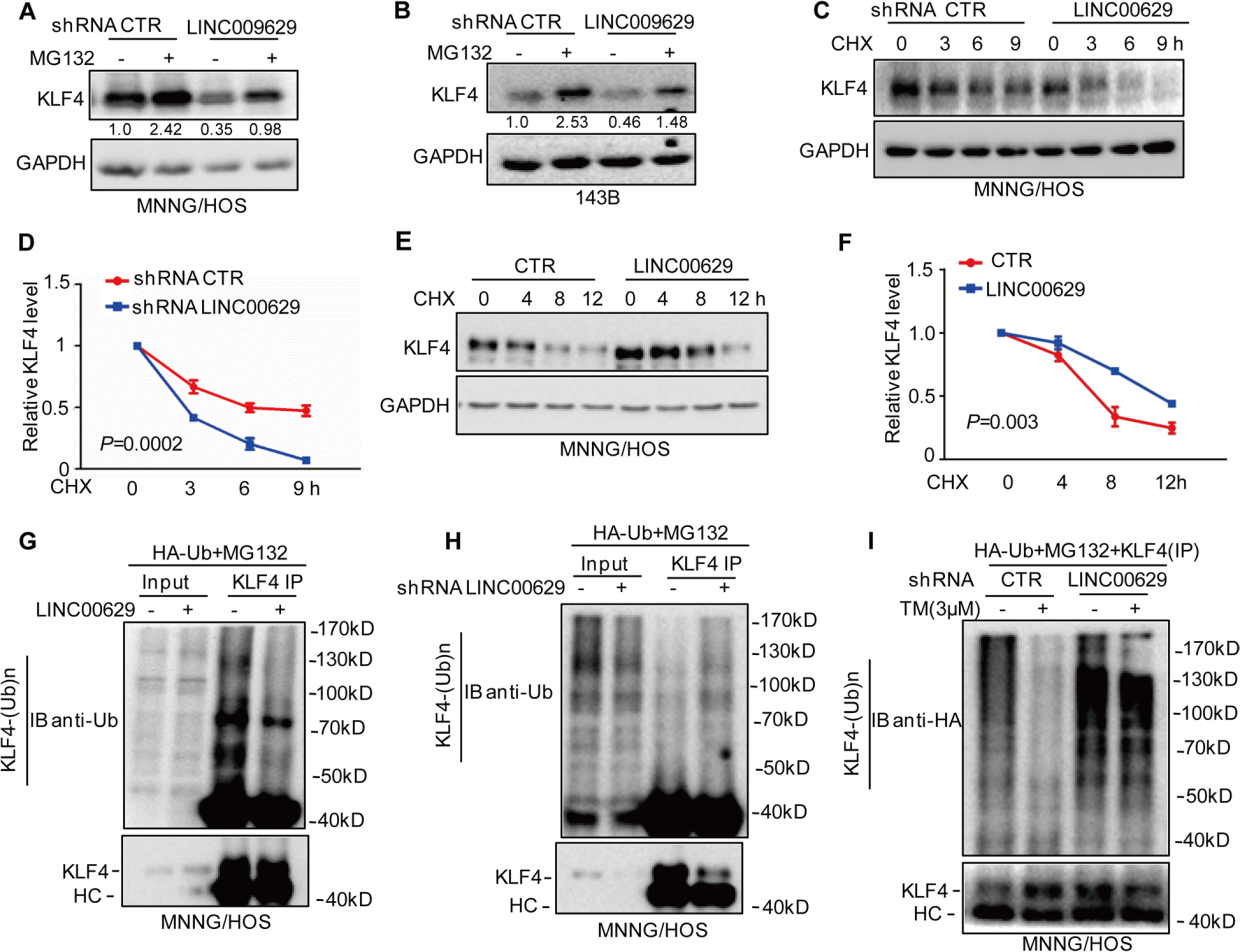
LINC00629 inhibits KLF4 degradation. **A**-**B** MNNG/HOS and 143B cells with or without LINC00629 knockdown were treated with 20 μM of the proteasome inhibitor MG132 for 8 h. The expression levels of KLF4 were detected by Western blot. Numbers represent the relative intensities of western blot bands of KLF4 to GAPDH. **C**-**D** MNNG/HOS and 143B cells with or without LINC00629 knockdown were treated with 10 mg/ml cycloheximide (CHX) for the indicated times. The expression levels of KLF4 were detected by Western blot (**C**), and the quantification of KLF4 levels relative to GAPDH is shown (**D**). The results are shown as the mean ± s.d. *n* = 3 independent experiments. *P* = 0.0002. **E**-**F**) MNNG/HOS cells with or without LINC00629 overexpression were treated with 10 mg/ml cycloheximide (CHX) for the indicated times. The expression levels of KLF4 were detected by Western blot (**E**), and the quantification of KLF4 levels relative to GAPDH is shown (**F**). The results are shown as the mean ± s.d. n = 3 independent experiments. *P* = 0.003. **G** MNNG/HOS cells with or without LINC00629 overexpression were transfected with the indicated constructs. After 24 h, the cells were treated with 20 μM MG132 for 8 h before collection. The whole-cell lysates were subjected to immunoprecipitation with KLF4 antibody and Western blot with anti-Ub antibody to detect ubiquitylated KLF4. **H** MNNG/HOS cells with or without LINC00629 knockdown were transfected with the indicated constructs. After 24 h, the cells were treated with 20 μM MG132 for 8 h before collection. The whole-cell lysates were subjected to immunoprecipitation with KLF4 antibody and Western blot with anti-Ub antibody to detect ubiquitylated KLF4. **I** MNNG/HOS cells with or without LINC00629 knockdown were transfected with the indicated constructs and treated with 3 μM TM. The cells were treated with 20 μM MG132 for 8 h before collection. The whole-cell lysates were subjected to immunoprecipitation with KLF4 antibody and Western blot with anti-HA antibody to detect ubiquitylated KLF4

To investigate whether LINC00629 enhanced KLF4 expression in a proteasome-dependent manner, MNNG/HOS and 143B cells were first treated with the proteasome inhibitor MG132, and the expression of KLF4 was analysed by Western blot. As shown in Fig. 4A-B, MG132 reversed the downregulation of KLF4 induced by LINC00629 knockdown. Subsequently, we treated these cells with the protein synthesis inhibitor CHX to assess the alteration of KLF4 half-life in LINC00629-depleted cells and found that inhibition of LINC00629 decreased the stability of endogenous KLF4 protein (Fig. 4E-F and Supplementary Fig. 4A-B). Conversely, overexpression of LINC00629 elevated the stability of KLF4 (Fig. 4E-F). Similarly, our further data indicated that elevated LINC000629 decreased the ubiquitination of KLF4 (Fig. 4G). Conversely, knockdown of LINC00629 increased the ubiquitination of KLF4 (Fig. 4H and Supplementary Fig. 4C).

To further investigate whether LINC00629 decreased the polyubiquitination of KLF4 under ER stress, MNNG/HOS cells with or without LINC00629 knockdown were treated with 3 μM TM as indicated, and the ubiquitination of KLF4 was detected by Western blot. As shown in Fig. 4I, ER stress decreased the ubiquitination of KLF4. However, the decrease was abolished by LINC00629 knockdown. Taken together, these results indicate that LINC00629 elevates the stability of KLF4 and inhibits its degradation.

### KLF4 transcriptionally upregulates LAMA4 expression

**Fig. 5:**
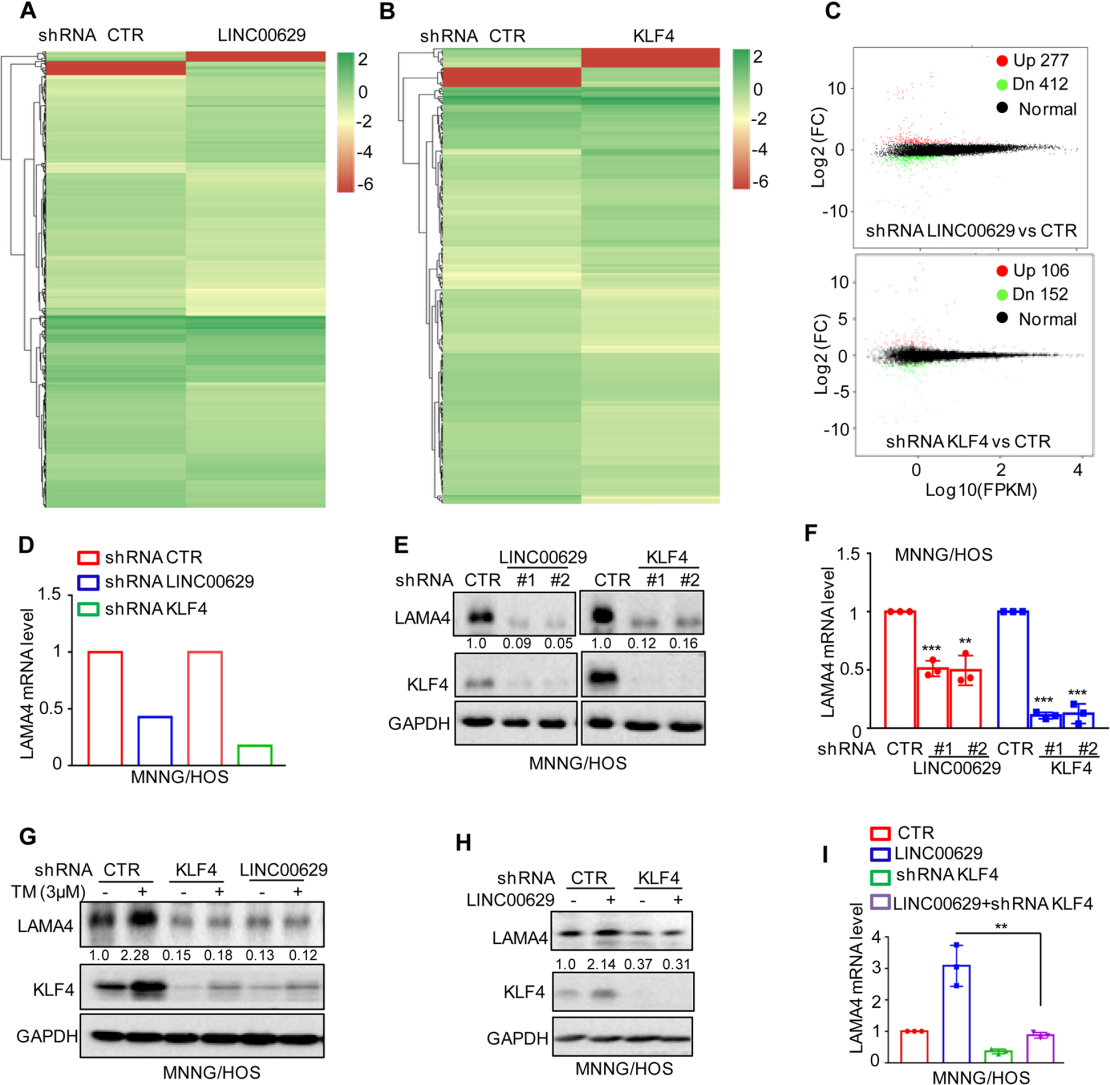
KLF4 upregulates LAMA4 expression. **A**-**B** Heatmap showing the altered genes identified by RNA sequencing analysis in LINC00629 (**A**)- or KLF4 (**B**)-depleted MNNG/HOS cells. **C** Volcano plots showing differentially expressed mRNAs for LINC00629 knockdown or KLF4 knockdown versus the controls (absolute log2-fold change > 1 and q value < 0.05). The green dot indicates the downregulated genes, and the red dot indicates the upregulated genes. **D** The mRNA levels of LAMA4 are shown from RNA sequencing data. **E**-**F** The protein and mRNA levels of LAMA4 were detected by Western blot (**E**) and qRT–PCR (**F**) in MNNG/HOS cells with or without LINC00629 or KLF4 knockdown. Numbers represent the relative intensities of western blot bands of LAMA4 to GAPDH. **G** MNNG/HOS cells with or without KLF4 or LINC00629 knockdown were treated with 3 μM TM for 36 h. The expression of LAMA4 was detected by Western blot. Numbers represent the relative intensities of western blot bands of LAMA4 to GAPDH. **H**-**I** LINC00629 was overexpressed in MNNG/HOS cells with or without KLF4 knockdown. The protein and mRNA levels of LAMA4 were detected by Western blotting (**H**) and qRT–PCR (**I**). Numbers represent the relative intensities of western blot bands of LAMA4 to GAPDH. Data in F and M were analysed by Student’s t test, *p < 0.05, **p < 0.01, ***p < 0.001

To identify the downstream genes of LINC00629 and KLF4, we used RNA sequencing to search for overmapping genes in MNNG/HOS cells with LINC00629 or KLF4 knockdown. We found 689 (277 upregulated and 412 downregulated) and 258 (106 upregulated and 152 downregulated) differentially expressed genes for LINC00629 and KLF4 knockdown, respectively (Fig. 5A-C and Supplementary Table 4 and 5). Not surprisingly, fifty genes exhibited overlapping expression (Supplementary Fig. 2A). Among these genes, we found that LAMA4 was significantly downregulated in LINC00629- and KLF4-depleted cells (Fig. 5D). To confirm this, the expression levels of LAMA4 were detected in LINC00629- or KLF4-depleted MNNG/HOS cells. Compared with the control cells, knockdown of LINC00629 and KLF4 notably suppressed LAMA4 expression (Fig. 5E-F). Similar results were obtained in 143B cells (Supplementary Fig. 5B-C). Additionally, we also found that inhibition of LINC00629 or KLF4 abolished ER stress-induced LAMA4 upregulation (Fig. 5G).

To investigate whether LINC00629 upregulated LAMA4 in a KLF4-dependent manner, we knocked down KLF4 in MNNG/HOS and 143B cells with or without LINC00629 overexpression. Then, the expression levels of LAMA4 were detected by Western blot and qRT–PCR. These results showed that depletion of KLF4 abolished the increase in LAMA4 by LINC00629 overexpression (Fig. 5H-I and Supplementary Fig. 5D-E). Collectively, our data indicate that LAMA4 is a downstream gene of the LINC00629-KLF4 pathway.

### KLF4 directly binds to the promoter of LAMA4

**Fig. 6:**
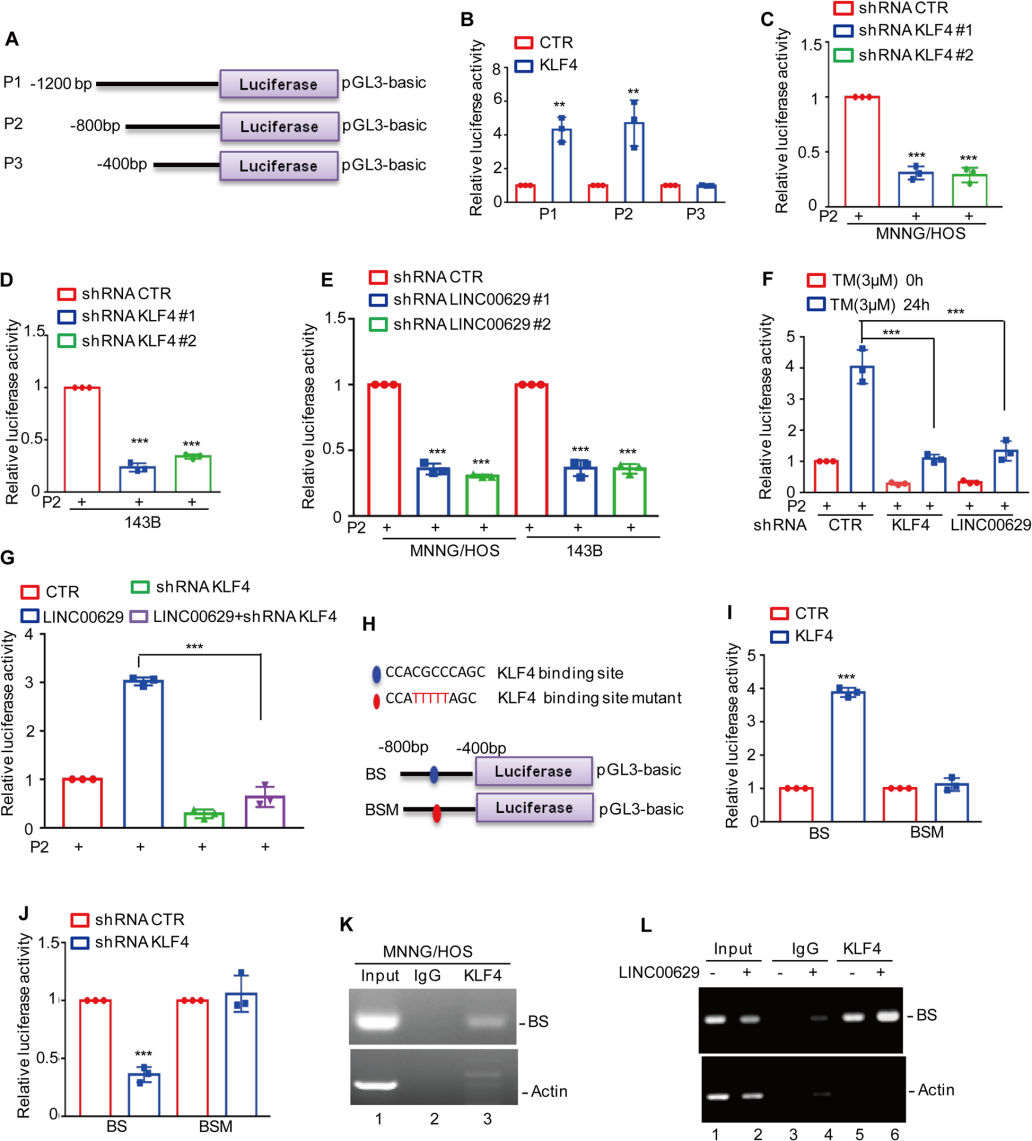
KLF4 binds to the promoter of LAMA4. **A** Schematic illustration of pGL3-based reporter constructs used in luciferase assays to examine the transcriptional activity of LAMA4 named P1, P2 and P3. **B** P1, P2 and P3 together with the *Renilla* luciferase plasmid were transfected into 293 T cells with or without KLF4 expression. The *Renilla* luciferase construct was used to control for transfection efficiency, and dual luciferase activity was measured. **C**-**D** P2 together with the *Renilla* luciferase plasmid was transfected into MNNG/HOS and 143B cells with or without KLF4 knockdown for 24 h. Dual luciferase activity was measured. **E** P2 together with the *Renilla* luciferase plasmid was transfected into MNNG/HOS and 143B cells with or without LINC00629 knockdown for 24 h, and the cells were collected. Dual luciferase activity was measured. **F** P2 together with the *Renilla* luciferase plasmid was transfected into MNNG/HOS cells with or without KLF4 or LINC00629 knockdown for 12 h, and the cells were then treated with 3 μM TM for another 24 h. Dual luciferase activity was measured. **G** LINC00629 was overexpressed in MNNG/HOS cells with or without KLF4 knockdown, and P2 together with the *Renilla* luciferase plasmid was transfected into the cells for 24 h. Dual luciferase activity was measured. **H** Schematic illustration of the KLF4 wild-type binding site (BS) and the matching mutant (BSM) that was used in luciferase assays. **I** The wild-type promoter (BS) or the matching mutant (BSM) together with the *Renilla* luciferase plasmid was individually transfected into MNNG/HOS cells with or without KLF4 overexpression. Dual luciferase activity was measured. **J** The wild-type promoter (BS) or the matching mutant (BSM) together with the *Renilla* luciferase plasmid was individually transfected into MNNG/HOS cells with or without KLF4 knockdown. Dual luciferase activity was measured. **K**-**L** ChIP analysis showed the binding of KLF4 to the promoter of LAMA4 in MNNG/HOS cells with or without LINC00629 overexpression. Isotype-matched IgG was used as a negative control. Data in B, C, D, E, F, G, I, J were analysed by Student’s t test, *p < 0.05, **p < 0.01, ***p < 0.001

To further confirm that KLF4 transcriptionally upregulated LAMA4, we assessed the effect of KLF4 on the promoter activity of LAMA4. The upstream sequence of LAMA4 and the truncations were cloned into pGL3-based luciferase reporter plasmids (named P1, P2, and P3), which were then transfected into 293 T cells with or without KLF4 overexpression (Fig. 6A). As shown in Fig. 6B, the luciferase activities of P1 and P2 but not P3 were increased in KLF4-overexpressing cells, suggesting that the region (− 800 to − 400 bp) was essential for KLF4-upregulated LAMA4 expression. Subsequently, we transfected P2 into MNNG/HOS and 143B cells with or without KLF4 or LINC00629 knockdown, and the luciferase activities of P2 were measured. As shown in Fig. 6C-E, depletion of KLF4 or LINC00629 decreased the luciferase activities of LAMA4 promoter (P2). Consistently, inhibition of KLF4 or LINC00629 abolished the ER stress-induced increase in the luciferase activities of LAMA4 promoter (Fig. 6F). To investigate whether LINC00629 affected the luciferase activity of P2 by upregulating KLF4, we then transfected P2 into LINC00629-overexpressing MNNG/HOS cells with or without KLF4 knockdown, and luciferase activity was measured. As expected, depletion of KLF4 eliminated the LAMA4 promoter luciferase activities increase by LINC00629 (Fig. 6G).

To further identify the potential binding sites of KLF4 on the LAMA4 promoter, we inspected the sequence of P2 using JASPAR software (https://jaspar.genereg.net), and one positive binding site was identified. To verify this hypothesis, we constructed two pGL3-based luciferase reporter plasmids containing a wild-type binding site (BS WT) and a mutant binding site (BS Mut) (Fig. 6H). These plasmids were individually transfected into 293 T cells with or without KLF4 overexpression, and the luciferase activity was measured. We found that the activity of BS WT but not BS Mut was significantly increased in response to KLF4 overexpression (Fig. 6I). Conversely, depletion of KLF4 decreased the luciferase activity of BS WT but not BS Mut (Fig. 6J).

Furthermore, the following chromatin immunoprecipitation (ChIP) assays showed that the chromatin fragment containing BS1 was specifically present in anti-KLF4 immunoprecipitation (Fig. 6K). The binding capacity of KLF4 to the LAMA4 promoter was enhanced in LINC00629-overexpressing MNNG/HOS cells (Fig. 6L). Taken together, these findings indicate that the BS region is of great significance for KLF4 to elevate LAMA4 expression and that LINC00629 upregulates LAMA4 in a KLF4-dependent manner.

### Knockdown of LAMA4 suppresses the malignant behaviour of osteosarcoma cell and accelerates ER stress-induced apoptosis

**Fig. 7:**
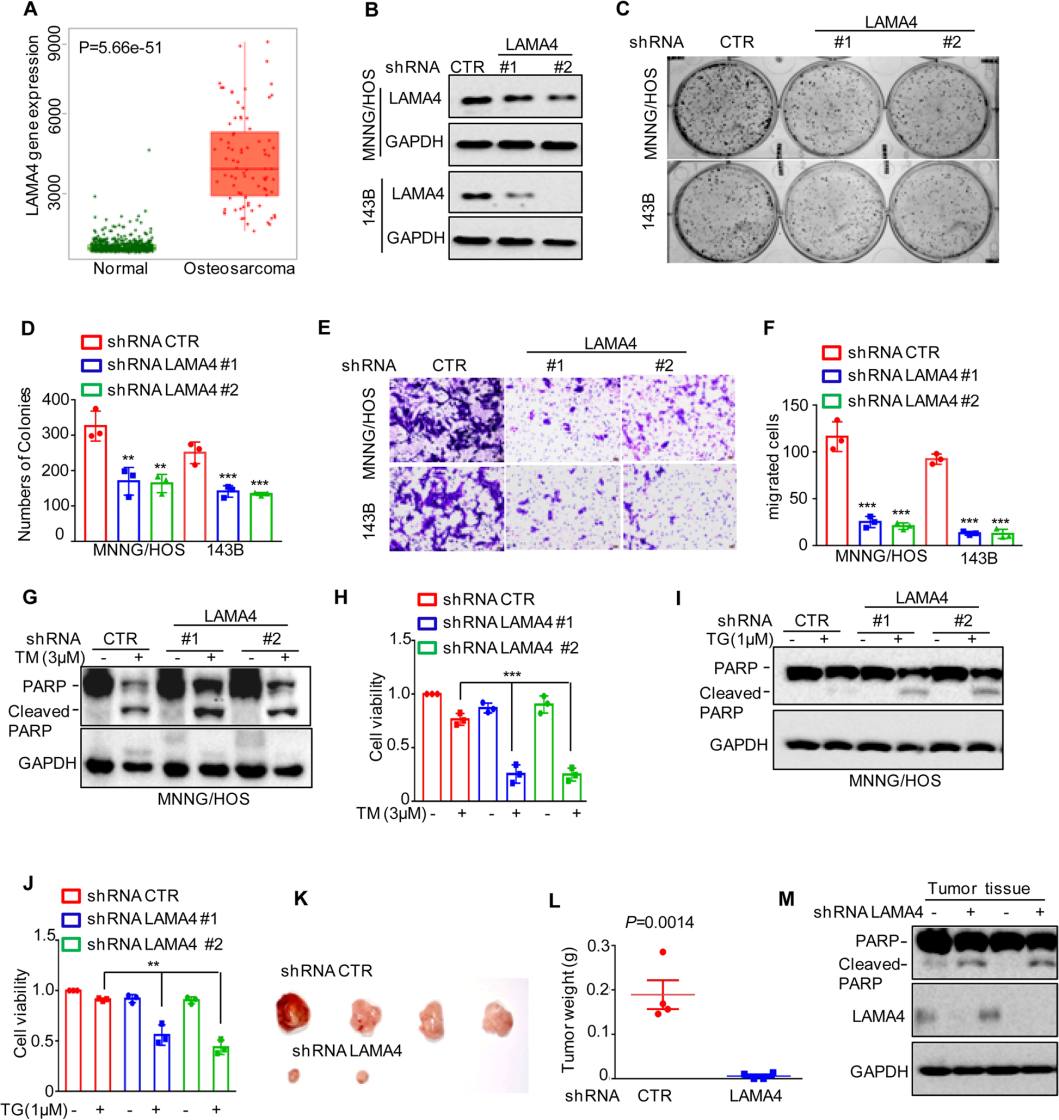
Knockdown of LAMA4 suppresses the malignant behaviour of osteosarcoma and accelerates ER stress-induced apoptosis. **A** The expression levels of LAMA4 in osteosarcoma (*n* = 88) and normal tissues (*n* = 564) were analysed from the TNMplot database. **B** LAMA4 was knocked down in MNNG/HOS and 143B cells. The expression of LAMA4 was detected by Western blot. GAPDH was used as the loading control. **C**-**D** The cells (3000 cells/well) with or without LAMA4 depletion were tested for cell growth in the colony formation assay. Viable colonies after 1 week were counted and are shown (**C**). Data are depicted as bar graphs (**D**). **E**-**F** The migration of the indicated cells was detected by Transwell assays. Representative images of crystal violet-stained culture plates are shown (**E**). Data are depicted as bar graphs (**F**). **G**-**H** MNNG/HOS cells with or without LAMA4 knockdown were treated with 3 μM TM for 36 h. Cell apoptosis and cell viability were analysed by Western blot (**G**) and CCK8 assays (**H**). GAPDH was used as the loading control. **I**-**J** MNNG/HOS cells with or without LAMA4 knockdown were treated with 1 μM TG for 36 h. Cell apoptosis and cell viability were analysed by Western blot (**I**) and CCK8 assays (**J**). GAPDH was used as the loading control. **K**-**M** MNNG/HOS cells (10^6^ cells per mouse) with or without LAMA4 knockdown were injected subcutaneously into nude mice (*n* = 4). Representative images of xenograft tumours (**K**). The weights of the tumours were calculated and analysed (L). The expression levels of PARP, cleaved PARP and LAMA4 in tumours were measured by Western blot (M). Data in D, F, H, J, L were analysed by Student’s t test, *p < 0.05, **p < 0.01, ***p < 0.001

LAMA4 belongs to the laminin family and plays an important role in many cancers [21]. However, its function in osteosarcoma is still unknown. To investigate this possibility, we first analysed the expression of LAMA4 from the TNMplot database and found that LAMA4 was significantly upregulated in osteosarcoma compared with normal tissues (Fig. 7A). To further evaluate the role of LAMA4 in osteosarcoma, we then knocked down LAMA4 in MNNG/HOS and 143B cells. As shown in Fig. 7B, compared with the control groups, the expression of LAMA4 in shRNA LAMA4 groups were substantially decreased. Then, Colony formation and Transwell assays were performed to measure the effects of LAMA4 on clonogenic potential and migration. As shown in Fig. 7C-F, depletion of LAMA4 markedly inhibited cell clonogenic potential and migration in MNNG/HOS and 143B cells, which was consistent with the role of KLF4 (Supplementary Fig. 6A-D). In addition, we also investigated the effects of LAMA4 on the cell adaptation to ER stress and found that knockdown of LAMA4 enhanced ER stress-induced apoptosis and promoted cell viability downregulation MNNG/HOS and 143B cells (Fig. 7G-J and Supplementary Fig. 6E-F).

To better understand the role of LAMA4 in osteosarcoma, a xenograft tumour formation assay was performed. MNNG/HOS cells with or without LAMA4 knockdown were injected into 4- to 6-week-old BALB/c (nu/nu) male nude mice. As shown in Fig. 7K-M, compared to the control group, inhibition of LAMA4 suppressed tumour growth, as indicated by a decrease in tumour weight, and accelerated cell apoptosis, as indicated by an increase in cleaved PARP. Taken together, our data indicate that LAMA4 plays an oncogenic role in MNNG/HOS and 143B cells.

### LINC00629 elevates the osteosarcoma cell adaption to ER stress and facilitates tumorigenesis by activating the KLF4-LAMA4 axis

**Fig. 8:**
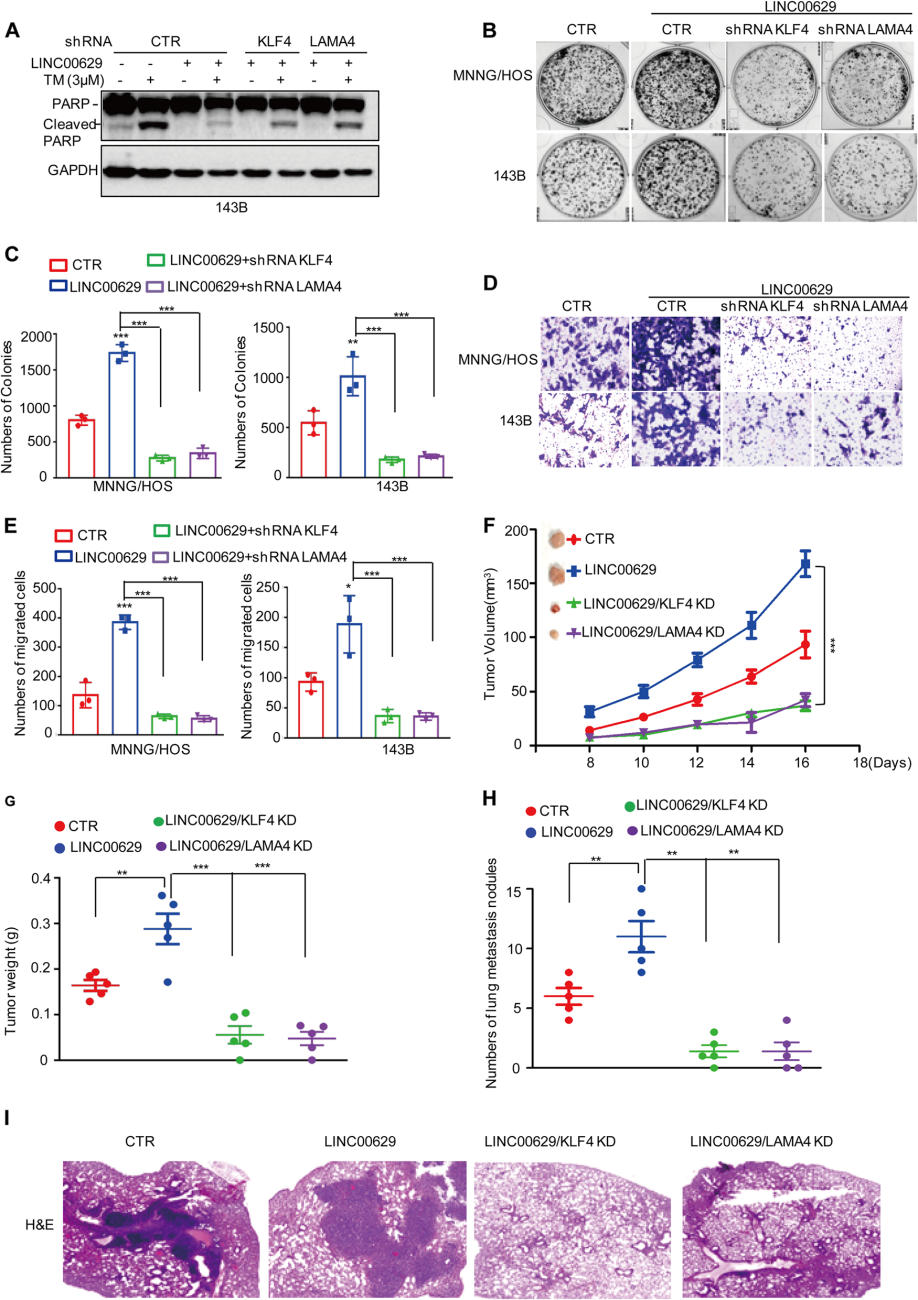
LINC00629 suppresses ER stress-induced apoptosis and facilitates osteosarcoma progression by activating the KLF4-LAMA4 axis. **A** KLF4 or LAMA4 was knocked down in 143B cells with or without LINC00629 overexpression. These cells were treated with 3 μM TM for 48 h, and cell apoptosis was detected by Western blot. GAPDH was used as a loading control. **B**-**C** KLF4 or LAMA4 was knocked down in MNNG/HOS and 143B cells with or without LINC00629 overexpression. The cells (3000 cells/well) were then tested for cell growth in the colony formation assay. Viable colonies after 1 week were counted and are shown (**B**). Data are depicted as bar graphs (**C**). **D**-**E** The migration of the indicated cells was detected by Transwell assays. Representative images of crystal violet-stained culture plates are shown (**D**). Data are depicted as bar graphs (**E**). **F**-**G** The shRNA KLF4 (KLF4 KD) or shRNA LAMA4 (LAMA4 KD) was used to knocked down in MNNG/HOS cells with or without LINC00629 overexpression. These cells (10^6^ cells per mouse) were injected subcutaneously into nude mice (*n* = 5). The volume (**F**) and weight (**G**) of the tumours were calculated and analysed. **H**-**I** The shRNA KLF4 (KLF4 KD) or shRNA LAMA4 (LAMA4 KD) was used to knocked down in MNNG/HOS cells with or without LINC00629 overexpression. These cells (10^6^ cells per mouse) were injected intravenously into nude mice (*n* = 5 per group). Representative images of HE staining are displayed (**I**). Each group of metastatic nodules was assessed (**H**). Data in C, E, F, G, H were analysed by Student’s t test, *p < 0.05, **p < 0.01, ***p < 0.001

To determine whether LINC00629 elevates the osteosarcoma cell adaption to ER stress and facilitates tumorigenesis by regulating the KLF4-LAMA4 axis, we first overexpressed LINC00629 in KLF4- or LAMA4-depleted MNNG/HOS cells and these cells were treated with 3 μM TM for 36 h. Cell apoptosis was detected by Western blot. The result showed that elevated LINC00629 suppressed ER stress-induced cell apoptosis, as indicated by the cleaved PARP increase. However, the inhibitory effect of LINC00629 on cell apoptosis was abolished when KLF4 or LAMA4 was knocked down (Fig. 8A). Similarly, we also observed that inhibition of KLF4 or LAMA4 eliminated the increase in clonogenicity and migration induced by LINC00629 overexpression (Fig. 8B-E).

To further confirm this hypothesis, xenograft tumour formation and metastasis assays were performed. We found that the promoting effects of LINC00629 on tumour development and lung metastasis were abolished by KLF4 or LAMA4 depletion in nude mice (Fig. 8F-I). Collectively, these results suggest that LINC00629 depends on the KLF4-LAMA4 axis to elevate the adaption to ER stress and promote tumour growth and metastasis in MNNG/HOS and 143B cells.

## Discussion

Metastatic osteosarcoma cells usually survive in nutritionally deprived and hypoxic environments, accompanied by prolonged ER stress. However, how osteosarcoma cells evade ER stress-induced apoptosis and survive under harsh conditions remains unclear. Here, we found that LINC00629 was increased in response to ER stress. Elevated LINC00629 promoted cell survival and facilitated tumorigenesis and metastasis by activating the KLF4-LAMA4 axis in MNNG/HOS and 143B cells.

LINC00629 is a long intergenic noncoding RNA mapped to chromosome X (Xq26). At present, only two studies reported the tumour suppressive role of LINC00629. One study indicated that LINC00629 suppressed the migration and invasion of JEG-3 cells [22]. Another study reported that LINC00629 suppressed tumour progression by upregulating AQP4 and competitively binding to miR-196b-5p in gastric cancer [23]. Interestingly, we showed that LINC00629 plays an oncogenic role in MNNG/HOS and 143B cells based on the following evidence. First, the ER stress promoted LINC00629 expression in MNNG/HOS and 143B cells. Furthermore, sarcoma patients with high levels of LINC00629 showed shorter overall survival than those with low levels of LINC00629. Second, knockdown of LINC00629 promoted ER stress-induced apoptosis and inhibited tumorigenesis and metastasis in vitro and in vivo. Whereas, overexpression of LINC00629 inhibited cell apoptosis and facilitated cell growth and metastasis. Except that, our further data indicated that LINC00629 upregulated KLF4 expression in MNNG/HOS and 143B cells.

KLF4 is a member of the KLF-like factor subfamily of zinc finger proteins, which play an ambivalent role in tumorigenesis as either a tumour suppressor or an oncogene in a number of cancers [24]. For example, KLF4 was reported to suppress hepatocellular carcinoma progression and inhibit gastric cancer cell proliferation by downregulating β-catenin expression [25, 26]. However, in breast cancer, melanoma, and glioma, KLF4 was shown to promote cell growth and inhibit cell apoptosis [27–29]. In osteosarcoma, KLF4 also enhanced tumorigenesis and promoted cell metastasis [30]. Previous studies indicated that the genetic context plays a decisive role in switching KLF4 between a tumour suppressor and a tumour promoter. For example, coexpression with an oncogenic RAS^*V12*^ allele or the downstream target gene cyclin D1 is sufficient to neutralize the cytostatic effects of KLF4 [31]. In osteosarcoma, some key oncogenes and key tumour suppressors, such as Kras or p53, usually exhibit mutations [32]. The information obtained from the Cellosaurs website (https://web.expasy.org/cellosaurus) indicated that MNNG/HOS and 143B also owned these mutation of the key genes which may determine the role of KLF4 in osteosarcoma and then leads to the switch of LINC00629 function in osteosarcoma cells.

KLF4 was also reported to be unstable and regulated by ubiquitination [33, 34]. The E3 ligases pVHL, FBXO32, Mule, and TRAF7 and the deubiquitinase USP10 are reported to promote KLF4 ubiquitination and degradation [35–39]. Similarly, our results suggested that LINC00629 was a novel lncRNA that interacted with KLF4 and inhibited its degradation in a proteasome-dependent manner.

Ultimately, our data showed that LAMA4 was a downstream gene of KLF4 and that deficiency of KLF4 or LINC00629 downregulated LAMA4 expression in MNNG/HOS and 143B cells. LAMA4 belongs to the laminin family and is primarily distributed in endothelial basement membranes and other tissues of mesodermal origin [40, 41]. Several reports have indicated that LAMA4 is regulated at different levels. For instance, miR-200b downregulated LAMA4 expression and suppressed renal cell carcinoma metastasis [42]. Androgen receptor (AR) or FOXM1 activated LAMA4 expression by directly binding to the promoter region [43, 44]. Consistently, we found that KLF4 also bound to the promoter region of LAMA4 and upregulated its expression in MNNG/HOS and 143B cells.

Increasing evidence indicates that LAMA4 plays a significant role in promoting survival, proliferation and migration in several cancers. For example, LAMA4 was highly expressed in breast cancer and promoted cell invasion [45]. Elevated LAMA4 promoted pancreatic cancer cell liver metastasis. In gastric cancer, LAMA4 is activated by the androgen receptor and enhances cell cisplatin resistance [44]. In osteosarcoma, we first reported that LAMA4 was upregulated in osteosarcoma and promoted proliferation and migration in MNNG/HOS and 143B cells. Subsequently, the correlation between LAMA4 expression and overall survival in sarcoma patients was obtained from the Kaplan–Meier plotter (KM plotter, http://kmplot.com) database. Unexpectedly, the data indicated that the patients with low LAMA4 mRNA expression had shorter overall survival, which was not consistent with the role of LAMA4 in MNNG/HOS and 143B cells. Thus, further studies are needed to investigate this possibility. Meanwhile, the potential mechanism whereby ER stress induced LINC00629 increase in osteosarcoma cells is still unknown and needs to be investigated in the future.

**Fig. 9:**
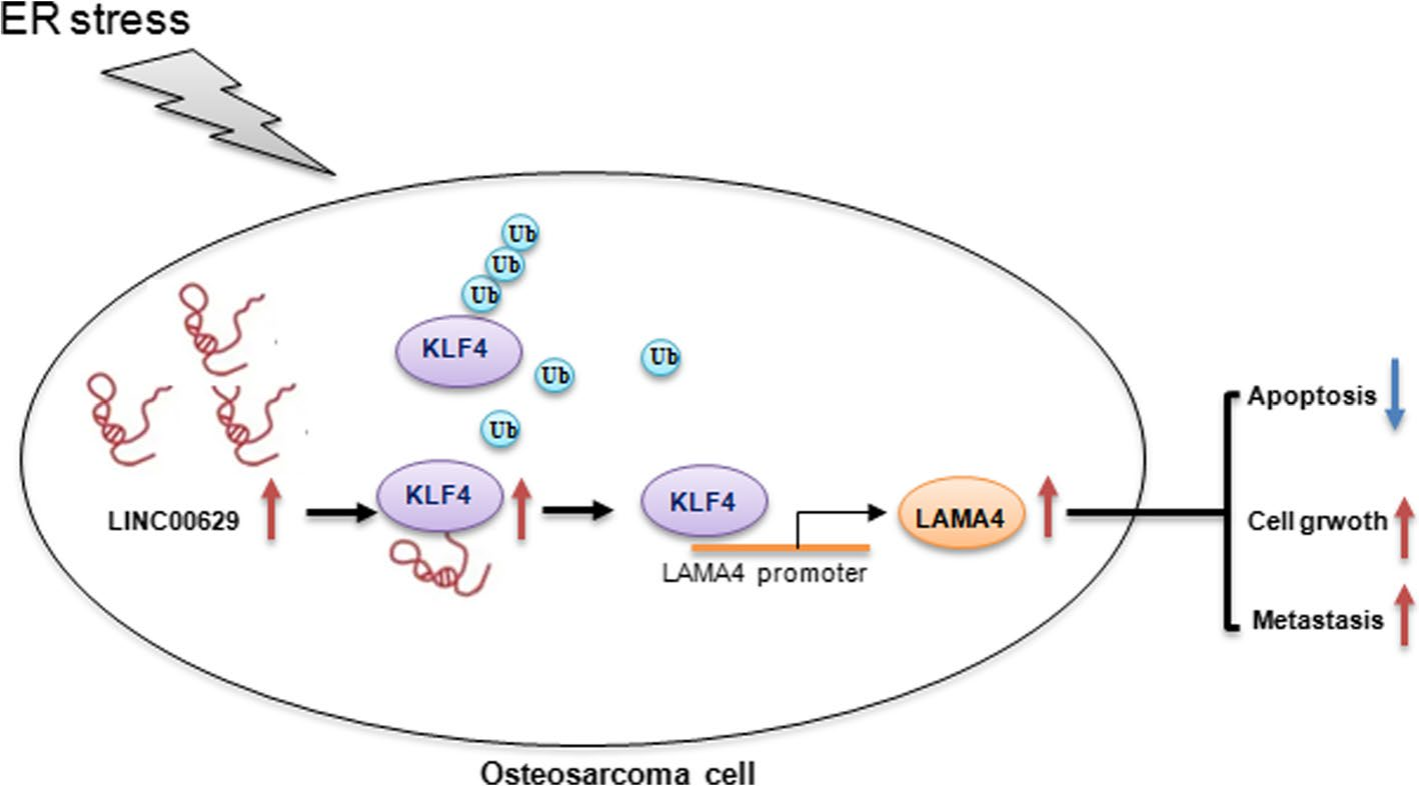
The schematic illustration of the role of LINC00629/KLF4/LAMA4 axis in ER stress-induced apoptosis and osteosarcoma progression

Collectively, our study suggests that ER stress induced LINC00629 expression, which in turn protected MNNG/HOS and 143B cells from ER stress-induced apoptosis by increasing KLF4 stability and inhibiting its degradation which then led to LAMA4 increase (Fig. 9).

## Conclusion

These findings demonstrate that LINC00629 is a key regulator of KLF4 and that the LINC00629-KLF4-LAMA4 axis plays an important role in the regulation of tumorigenesis and metastasis in MNNG/HOS and 143B cells.

## Supplementary Information

**Additional file 1: Figure S1.** (A) 143B cells were treated with 3 μM TM for 0, 24 and 36 h. The expression levels of LINC00629 were detected by qRT-PCR. (B) Kaplan–Meier plot of the overall survival rate of 259 patients with sarcoma. The data were obtained from the Kaplan-Meier Plotter. (C) LINC00629 was knocked down in MNNG/HOS and143B cells using lentivirus expressing shRNAs. The expression of LINC00629 was detected by qRT-PCR. (D-E) 143B cells with or without LINC00629 knockdown were treated with or without 3 μM TM for 36 h. Cell apoptosis were detected by Western blot (D) and cell viability was analyzed by CCK8 assay (E). GRP78 was used as the ER stress marker and GAPDH was used as loading control. (F) LINC00629 was overexpressed in 143B cells using lentivirus expressing pCDH vector. The expression of LINC00629 was detected by qRT-PCR. (G) The siRNAPool for LINC00629 was transfected into MNNG/HOS cells. After 36 h, the cells were collected and the expression of LINC00629 was analyzed by qRT-PCR. (H-I) MNNG/HOS cells with or without LINC00629 knockdown using siRNAPool were treated with or without 3 μM TM for 36 h. Cell apoptosis were detected by Western blot (H) and cell viability was analyzed by CCK8 assay (I). GRP78 was used as the ER stress marker and GAPDH was used as loading control. (J) The relative expression of LINC00629 was detected in SJSA-1, U2OS, 143B, MNNG/HOS and MG63 cells. (K) SJSA-1 cells were treated with 3 μM TM for 0, 12 and 24 h. Cell apoptosis were detected by Western blot. (L) SJSA-1 cells with or without LINC00629 overexpression were treated with 3 μM TM for 24 h. Cell apoptosis were detected by Western blot. Data in A, C, E, F, G and I were analysed by Student’s t-test, *p < 0.05, **p < 0.01, ***p < 0.001.

**Additional file 2: Figure S2.** (A-B) The osteosarcoma cells (3000 cell/well) with or without LINC00629 overexpression were tested for the cell growth in the colony formation assay. Viable colonies after 1 week were counted and were shown(A). Data are depicted as bar graphs (B). (C-D) The migration of the indicated cells was detected by Transwell assays. Represented images of crystal violet-stained culture plates were shown (C). Data are depicted as bar graphs (D). Data in B and D were analysed by Student’s t-test, *p < 0.05, **p < 0.01, ***p < 0.001.

**Additional file 3: Figure S3.** (A-B) The mRNA levels of KLF4 were detected by qRT-PCR in MNNG/HOS and 143B cells with or without LINC00629 knockdown. (C) 143B cells with or without LINC00629 knockdown were treated with 3 μM TM for 36 h. The expression levels of KLF4 were detected by Western blot. Numbers represent the relative intensities of western blot bands of KLF4 to GAPDH. (D) KLF4 antibody (2 μg) was used to coprecipitate with LINC00629 in whole –cell lysates of 143B cells. The levels of LINC00629 were detected by RT-PCR and KLF4 protein levels were analyzed by Western blot using KLF4 antibody. (E) KLF4 antibody was used to coprecipitate with LINC00629 in whole –cell lysates of 143B cells with or without 3 μM TM treatment. The levels of LINC00629 were detected by RT-PCR and KLF4 protein levels were analyzed by Western blot using KLF4 antibody.

**Additional file 4: Figure S4.** (A-B) 143B cells with or without LINC00629 knockdown were treated with 10 mg/ml cycloheximide (CHX) for the indicated times. The expression levels of KLF4 were detected by Western blot (A) and quantification of KLF4 levels relative to GAPDH is shown (B). Results are shown as mean ± s.d. *n* = 3 independent experiments. *P* = 0.003. (C) The 143B cells with or without LINC00629 knockdown were transfected with the indicated constructs. After 24 h, the cells were treated with 20 μM MG132 for 8 h before collection. The whole-cell lysates were subjected to immunoprecipitation with KLF4 antibody and Western blot with anti-Ub antibody to detect ubiquitylated KLF4.

**Additional file 5: Figure S5.** (A) Overlaps indicating numbers of the differentially expressed mRNAs between KLF4 Knockdown and LINC00629 knockdown conditions. (B-C) The protein and mRNA levels of LAMA4 were detected by Western blot (B) and qRT-PCR (C) in 143B cells with or without LINC00629 or KLF4 knockdown. Numbers represent the relative intensities of western blot bands of LAMA4 to GAPDH. (D-E) LINC00629 was overexpressed in 143B cells with or without KLF4 knockdown. The protein and mRNA levels of LAMA4 were detected by Western blot (D) and qRT-PCR (E). Numbers represent the relative intensities of western blot bands of LAMA4 to GAPDH. Data in C, and E were analyzed by Student’s t-test, *p < 0.05, **p < 0.01, ***p < 0.001.

**Additional file 6: Figure S6.** (A-B) The osteosarcoma cells (3000 cell/well) with or without KLF4 knockdown were tested for the cell growth in the colony formation assay. Viable colonies after 1 week were counted and were shown (A). Data are depicted as bar graphs (B). (C-D) The migration of the indicated cells was detected by transwell assays. Represented images of crystal violet-stained culture plates were shown (C). Data are depicted as bar graphs (D). (E-F) 143B cells with or without LAMA4 knockdown were treated with 3 μM TM for 36 h. Cell apoptosis and cell viability was analysed by Western blot (E) and CCK8 assays (F). GAPDH was used as the loading control. (G-H) 143B cells with or without LAMA4 knockdown were treated with 1 μM TG for 36 h. Cell apoptosis and cell viability was analysed by Western blot (G) and CCK8 assays (H). GAPDH was used as the loading control. (I) Kaplan–Meier plot of the overall survival rate of 269 patients with sarcoma. The data were obtained from the Kaplan-Meier Plotter. Data in B, D, and F were analyzed by Student’s t-test, *p < 0.05, **p < 0.01, ***p < 0.001.

**Additional file 7: Supplementary Table 1.** The shRNAs used in the article.

**Additional file 8: Supplementary Table 2.** The primers used in the article.

**Additional file 9: Supplementary Table 3.** The altered LncRNAs under 3 μM TM treatment.

**Additional file 10: Supplementary Table 4.** The altered genes in LINC00629-depleted cells.

**Additional file 11: Supplementary Table 5.** The altered genes in KLF4-depleted cells.

## Abbreviations

ER stress: Endoplasmic reticulum stress
TM: Tunicamycin
ChIP: Chromatin Immunoprecipitation
qRT-PCR: quantitative real-time polymerase chain reaction assay
CCK8: Cell Counting Kit-8
LAMA4: Laminin subunit alpha 4. LncRNA: Long non-coding RNA
