# Supplementary figures and images for: LINC00629 protects osteosarcoma cell from ER stress-induced apoptosis and facilitates tumour progression by elevating KLF4 stability
Source: J Exp Clin Cancer Res. 2022 Dec 20;41:354. doi: 10.1186/s13046-022-02569-x (PMC9764730; doi:10.1186/s13046-022-02569-x)

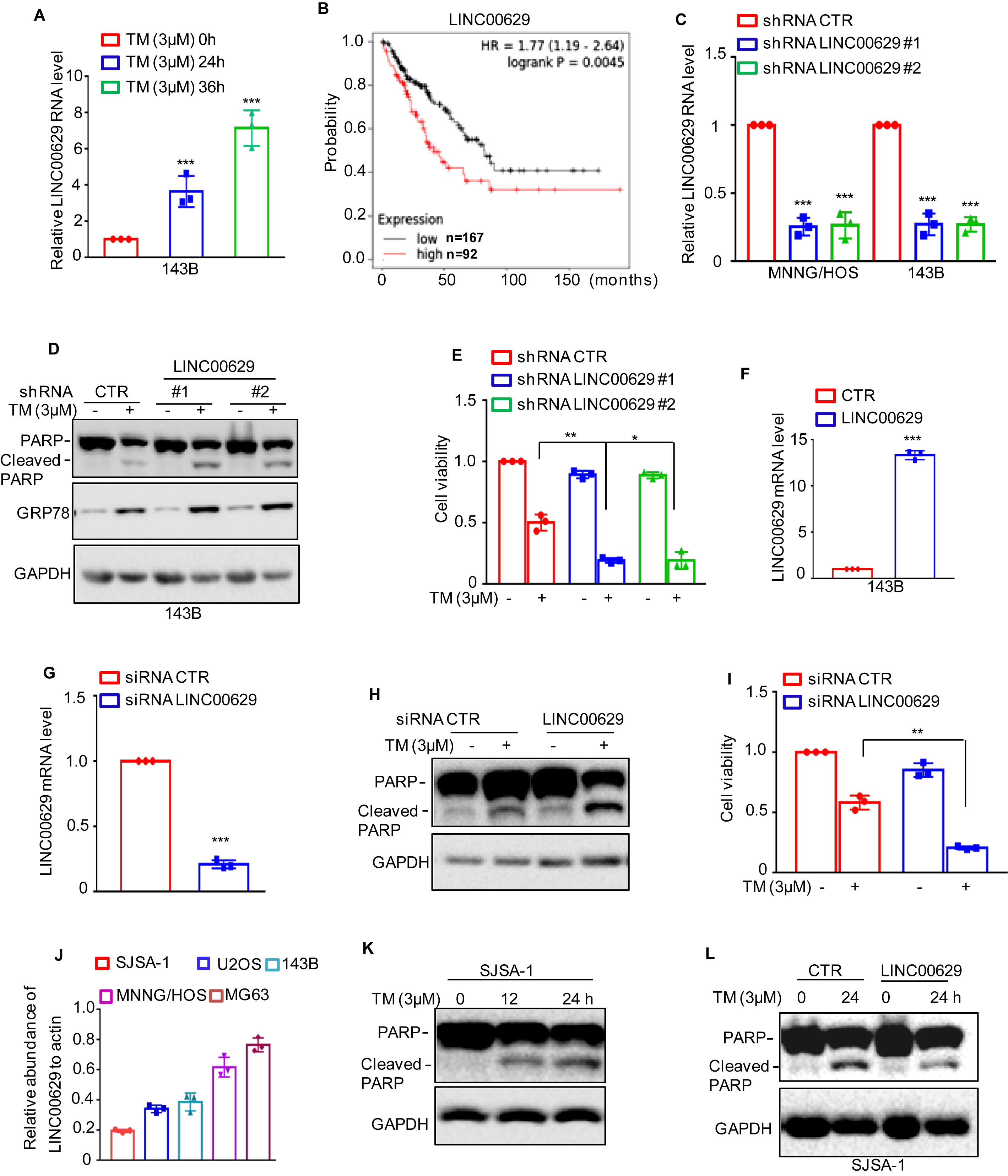

Supplement: Supplementary file 1 — Additional file 1: Figure S1. (A) 143B cells were treated with 3 μM TM for 0, 24 and 36 h. The expression levels of LINC00629 were detected by qRT-PCR. (B) Kaplan–Meier plot of the overall survival rate of 259 patients with sarcoma. The data were obtained from the Kaplan-Meier Plotter. (C) LINC00629 was knocked down in MNNG/HOS and143B cells using lentivirus expressing shRNAs. The expression of LINC00629 was detected by qRT-PCR. (D-E) 143B cells with or without LINC00629 knockdown were treated with or without 3 μM TM for 36 h. Cell apoptosis were detected by Western blot (D) and cell viability was analyzed by CCK8 assay (E). GRP78 was used as the ER stress marker and GAPDH was used as loading control. (F) LINC00629 was overexpressed in 143B cells using lentivirus expressing pCDH vector. The expression of LINC00629 was detected by qRT-PCR. (G) The siRNAPool for LINC00629 was transfected into MNNG/HOS cells. After 36 h, the cells were collected and the expression of LINC00629 was analyzed by qRT-PCR. (H-I) MNNG/HOS cells with or without LINC00629 knockdown using siRNAPool were treated with or without 3 μM TM for 36 h. Cell apoptosis were detected by Western blot (H) and cell viability was analyzed by CCK8 assay (I). GRP78 was used as the ER stress marker and GAPDH was used as loading control. (J) The relative expression of LINC00629 was detected in SJSA-1, U2OS, 143B, MNNG/HOS and MG63 cells. (K) SJSA-1 cells were treated with 3 μM TM for 0, 12 and 24 h. Cell apoptosis were detected by Western blot. (L) SJSA-1 cells with or without LINC00629 overexpression were treated with 3 μM TM for 24 h. Cell apoptosis were detected by Western blot. Data in A, C, E, F, G and I were analysed by Student’s t-test, *p < 0.05, **p < 0.01, ***p < 0.001. [file 13046_2022_2569_MOESM1_ESM.tif]

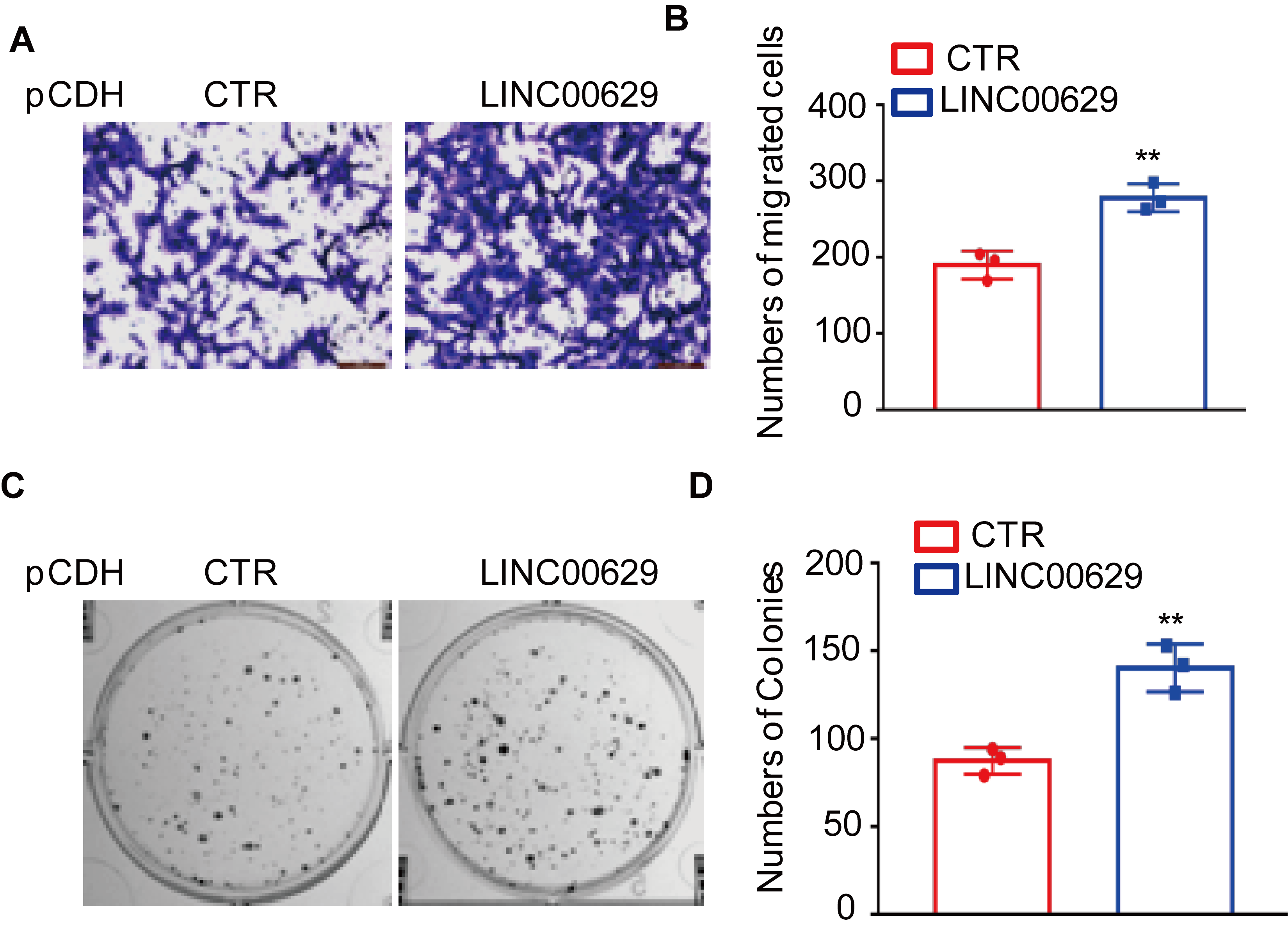

Supplement: Supplementary file 2 — Additional file 2: Figure S2. (A-B) The osteosarcoma cells (3000 cell/well) with or without LINC00629 overexpression were tested for the cell growth in the colony formation assay. Viable colonies after 1 week were counted and were shown(A). Data are depicted as bar graphs (B). (C-D) The migration of the indicated cells was detected by Transwell assays. Represented images of crystal violet-stained culture plates were shown (C). Data are depicted as bar graphs (D). Data in B and D were analysed by Student’s t-test, *p < 0.05, **p < 0.01, ***p < 0.001. [file 13046_2022_2569_MOESM2_ESM.tif]

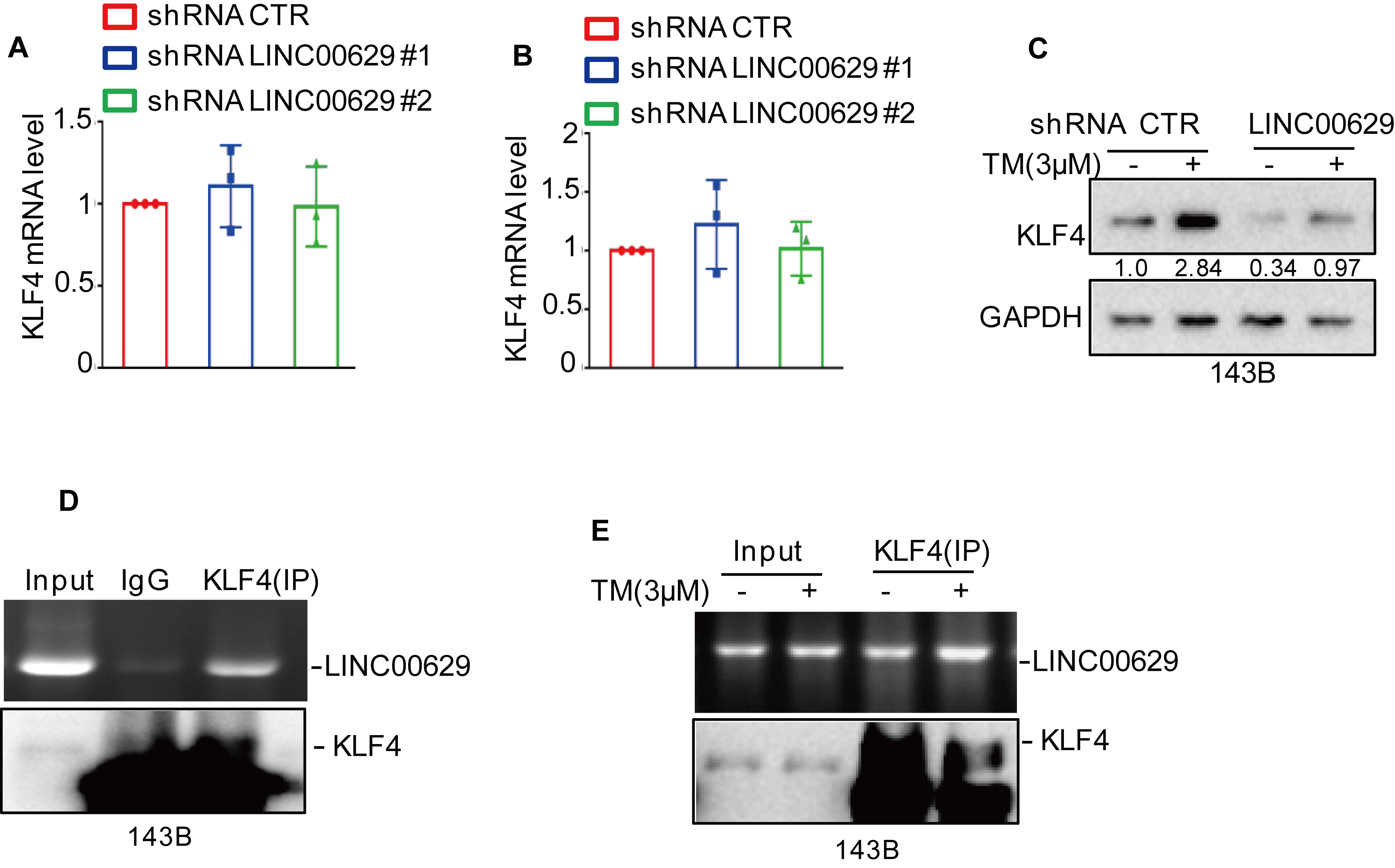

Supplement: Supplementary file 3 — Additional file 3: Figure S3. (A-B) The mRNA levels of KLF4 were detected by qRT-PCR in MNNG/HOS and 143B cells with or without LINC00629 knockdown. (C) 143B cells with or without LINC00629 knockdown were treated with 3 μM TM for 36 h. The expression levels of KLF4 were detected by Western blot. Numbers represent the relative intensities of western blot bands of KLF4 to GAPDH. (D) KLF4 antibody (2 μg) was used to coprecipitate with LINC00629 in whole –cell lysates of 143B cells. The levels of LINC00629 were detected by RT-PCR and KLF4 protein levels were analyzed by Western blot using KLF4 antibody. (E) KLF4 antibody was used to coprecipitate with LINC00629 in whole –cell lysates of 143B cells with or without 3 μM TM treatment. The levels of LINC00629 were detected by RT-PCR and KLF4 protein levels were analyzed by Western blot using KLF4 antibody. [file 13046_2022_2569_MOESM3_ESM.tif]

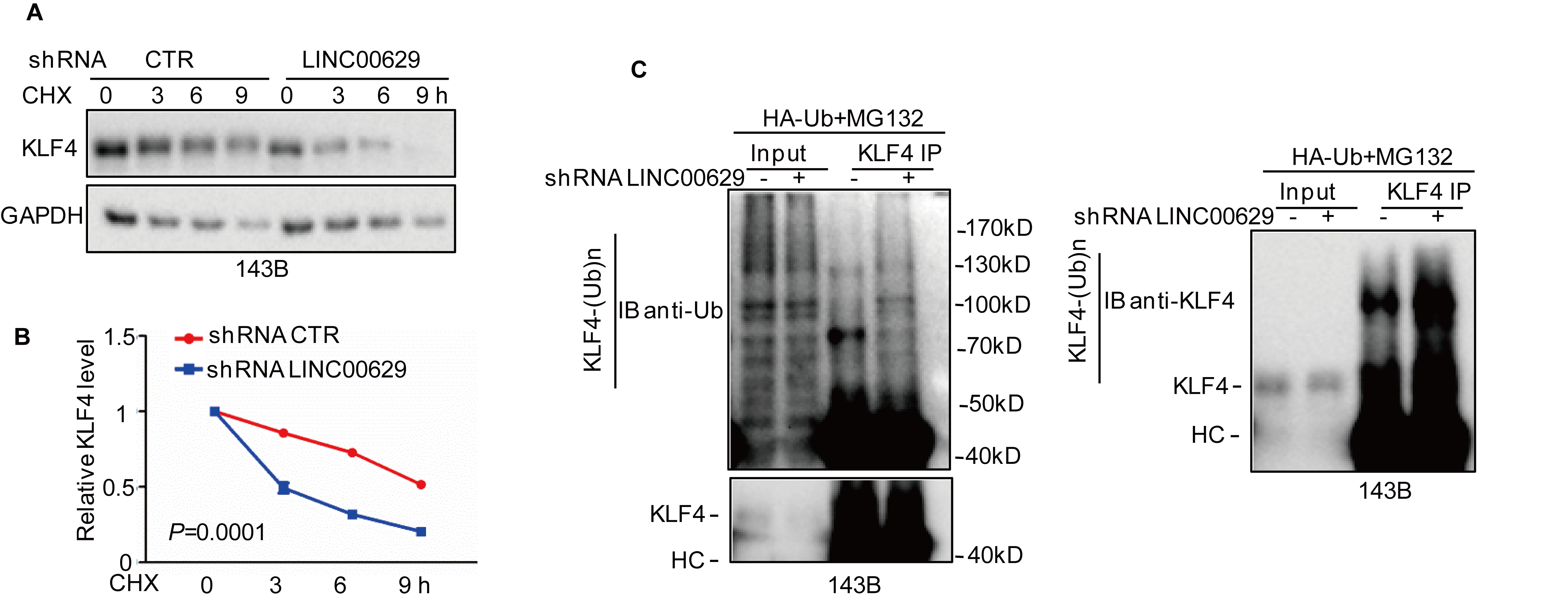

Supplement: Supplementary file 4 — Additional file 4: Figure S4. (A-B) 143B cells with or without LINC00629 knockdown were treated with 10 mg/ml cycloheximide (CHX) for the indicated times. The expression levels of KLF4 were detected by Western blot (A) and quantification of KLF4 levels relative to GAPDH is shown (B). Results are shown as mean ± s.d. n = 3 independent experiments. P = 0.003. (C) The 143B cells with or without LINC00629 knockdown were transfected with the indicated constructs. After 24 h, the cells were treated with 20 μM MG132 for 8 h before collection. The whole-cell lysates were subjected to immunoprecipitation with KLF4 antibody and Western blot with anti-Ub antibody to detect ubiquitylated KLF4. [file 13046_2022_2569_MOESM4_ESM.tif]

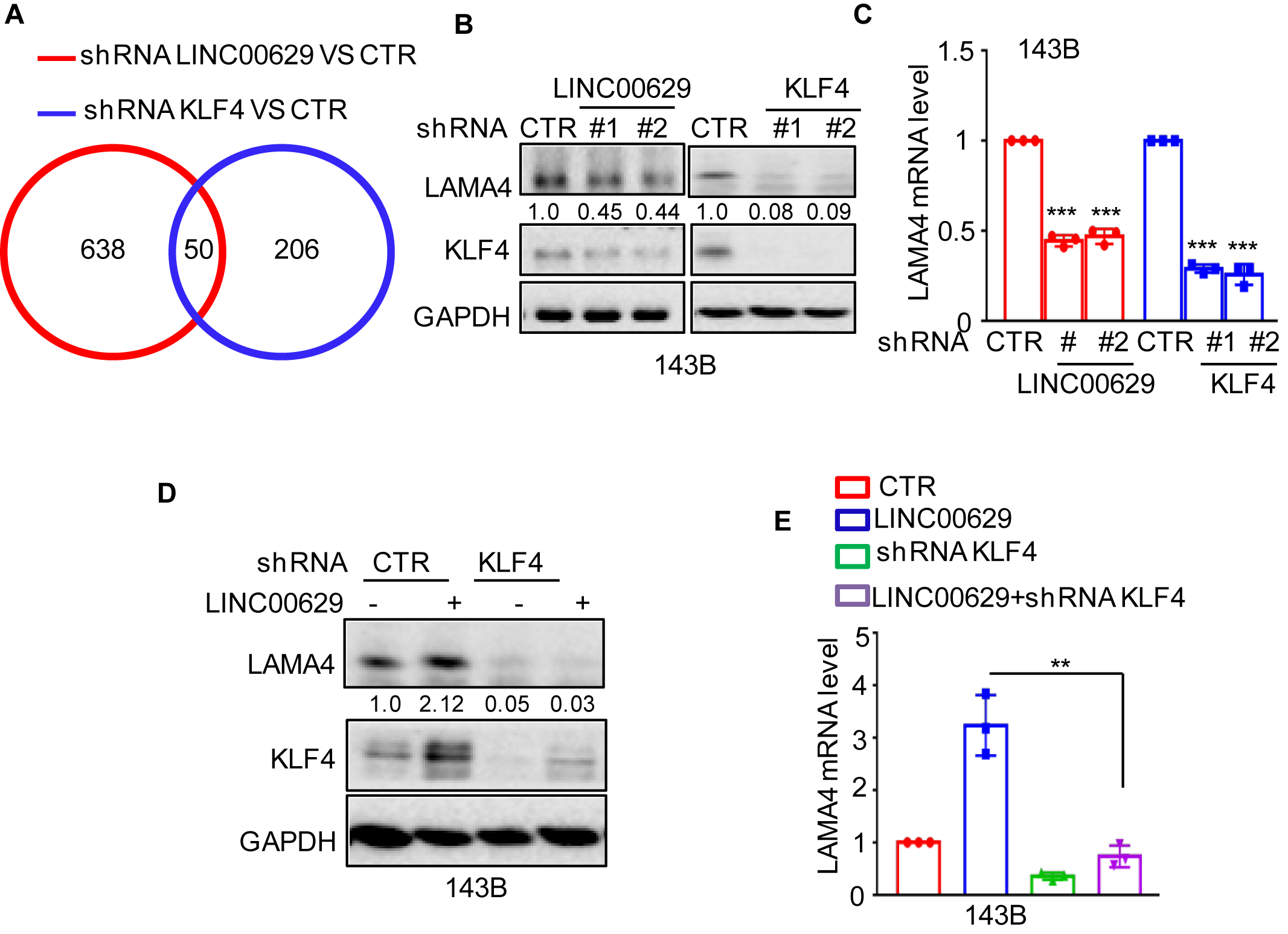

Supplement: Supplementary file 5 — Additional file 5: Figure S5. (A) Overlaps indicating numbers of the differentially expressed mRNAs between KLF4 Knockdown and LINC00629 knockdown conditions. (B-C) The protein and mRNA levels of LAMA4 were detected by Western blot (B) and qRT-PCR (C) in 143B cells with or without LINC00629 or KLF4 knockdown. Numbers represent the relative intensities of western blot bands of LAMA4 to GAPDH. (D-E) LINC00629 was overexpressed in 143B cells with or without KLF4 knockdown. The protein and mRNA levels of LAMA4 were detected by Western blot (D) and qRT-PCR (E). Numbers represent the relative intensities of western blot bands of LAMA4 to GAPDH. Data in C, and E were analyzed by Student’s t-test, *p < 0.05, **p < 0.01, ***p < 0.001. [file 13046_2022_2569_MOESM5_ESM.tif]

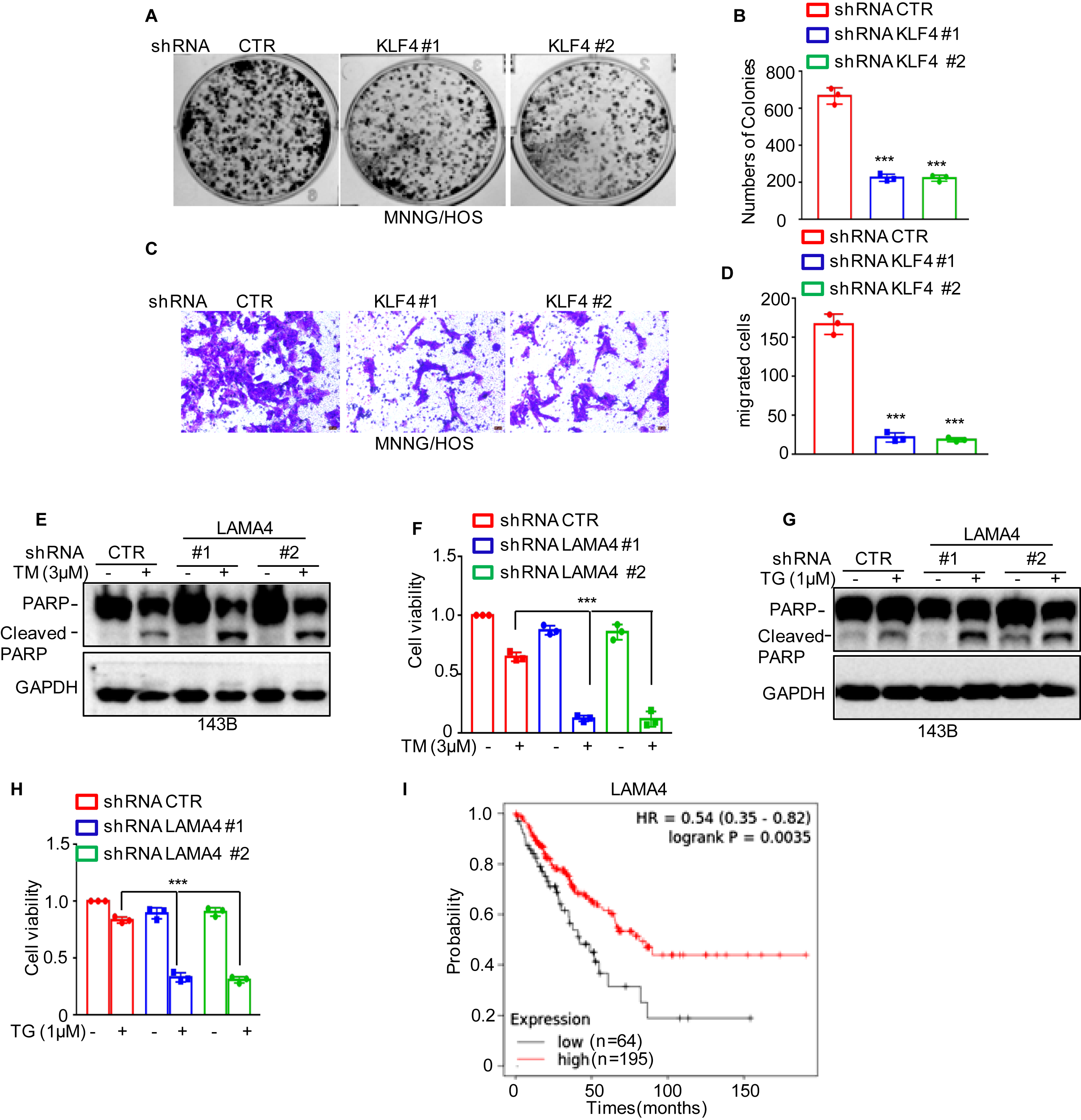

Supplement: Supplementary file 6 — Additional file 6: Figure S6. (A-B) The osteosarcoma cells (3000 cell/well) with or without KLF4 knockdown were tested for the cell growth in the colony formation assay. Viable colonies after 1 week were counted and were shown (A). Data are depicted as bar graphs (B). (C-D) The migration of the indicated cells was detected by transwell assays. Represented images of crystal violet-stained culture plates were shown (C). Data are depicted as bar graphs (D). (E-F) 143B cells with or without LAMA4 knockdown were treated with 3 μM TM for 36 h. Cell apoptosis and cell viability was analysed by Western blot (E) and CCK8 assays (F). GAPDH was used as the loading control. (G-H) 143B cells with or without LAMA4 knockdown were treated with 1 μM TG for 36 h. Cell apoptosis and cell viability was analysed by Western blot (G) and CCK8 assays (H). GAPDH was used as the loading control. (I) Kaplan–Meier plot of the overall survival rate of 269 patients with sarcoma. The data were obtained from the Kaplan-Meier Plotter. Data in B, D, and F were analyzed by Student’s t-test, *p < 0.05, **p < 0.01, ***p < 0.001. [file 13046_2022_2569_MOESM6_ESM.tif]
